# Electronic and Structural Transitions of LaAlO_3_/SrTiO_3_ Heterostructure Driven by Polar Field‐Assisted Oxygen Vacancy Formation at the Surface

**DOI:** 10.1002/advs.202002073

**Published:** 2021-05-24

**Authors:** Kyung Song, Taewon Min, Jinsol Seo, Sangwoo Ryu, Hyungwoo Lee, Zhipeng Wang, Si‐Young Choi, Jaekwang Lee, Chang‐Beom Eom, Sang Ho Oh

**Affiliations:** ^1^ Materials Testing and Reliability Division Korea Institute of Materials Science (KIMS) Changwon 51508 Republic of Korea; ^2^ Department of Materials Science and Engineering Pohang University of Science and Technology (POSTECH) Pohang 37673 Republic of Korea; ^3^ Department of Physics Pusan National University Busan 46241 Republic of Korea; ^4^ Department of Energy Science Sungkyunkwan University Suwon 16419 Republic of Korea; ^5^ Department of Materials Science and Engineering University of Wisconsin‐Madison Madison WI 53706 USA

**Keywords:** 2D electron gas, antiferrodistortive rotation, LaAlO_3_/SrTiO_3_, oxygen vacancy, STEM

## Abstract

The origin of 2D electron gas (2DEG) at LaAlO_3_/SrTiO_3_ (LAO/STO) interfaces has remained highly controversial since its discovery. Various models are proposed, which include electronic reconstruction via surface‐to‐interface charge transfer and defect‐mediated doping involving cation intermixing or oxygen vacancy (*V*_O_) formation. It is shown that the polar field‐assisted *V*_O_ formation at the LAO/STO surface plays critical roles in the 2DEG formation and concurrent structural transition. Comprehensive scanning transmission electron microscopy analyses, in conjunction with density functional theory calculations, demonstrate that *V*_O_ forming at the LAO/STO surface above the critical thickness (*t*_c_) cancels the polar field by doping the interface with 2DEG. The antiferrodistortive (AFD) octahedral rotations in LAO, which are suppressed below the *t*_c_, evolve with the formation of *V*_O_ above the *t*_c_. The present study reveals that local symmetry breaking and shallow donor behavior of *V*_O_ induce the AFD rotations and relieve the electrical field by electron doping the oxide heterointerface.

## Graphical abstract

Comprehensive scanning transmission electron microscopy analyses, in conjunction with density functional theory calculations, demonstrate that oxygen vacancy forming at LaAlO_3_/SrTiO_3_ (001) surface above the critical thickness (*t*_c_) cancels the polar electric field by doping the interface with two‐dimensional electron gas. The antiferrodistortive octahedral rotations in LaAlO_3_, which are suppressed below *t*_c_, evolve with the formation of oxygen vacancy above *t*_c_.

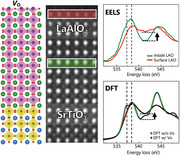

The polar discontinuity across an atomically controlled polar/nonpolar oxide heterostructure derives the orbital reconstruction and quantum confinement of electrons at the interface, which endows emergent physical properties absent in the bulk oxides.^[^ ^1^, ^2^ ^]^ A notable example is LaAlO_3_/SrTiO_3_ (LAO/STO) heterointerface, where the atomic stacking sequence changes from the charge‐neutral layers (SrO/TiO_2_) of STO to the charged layers (AlO_2_^−^/LaO^+^) of LAO.^[^ ^3^, ^4^ ^]^ If not compensated, the polar electric field in the LAO would grow with its thickness. The electrostatic instability set in by this electric field is relieved by forming 2D electron gas (2DEG) in the STO side of interface at the critical thickness (*t*_c_) of LAO (4 unit cell, u.c.).^[^ ^5^, ^6^ ^]^ Regarding the formation mechanism of the 2DEG, various models have been proposed based on different electron sources; these include the electronic reconstruction via surface‐to‐interface charge transfer^[^ ^3^, ^4^ ^]^ and defect‐mediated doping involving either cation intermixing^[^ ^7^, ^8^, ^9^ ^]^ or oxygen vacancy (*V*_O_) formation,^[^ ^10^, ^11^, ^12^ ^]^ each of which has been validated on its own theoretical foundation.^[^ ^8^, ^13^, ^14^ ^]^

The origin of the 2DEG is still a lively debating subject. Initially, based on an ideal defect‐free interface model, the intrinsic doping by electronic reconstruction was proposed^[^ ^3^ ^]^ and validated by density functional theory (DFT) calculation^[^ ^15^ ^]^—the diverging potential that grows with LAO thickness until the LAO valence band maximum crosses the Fermi level of the STO gives rise to electron transfer from the LAO surface to the interface. The transferred electrons compensate the diverging potential and stabilize the system. However, the so‐called polar catastrophe model has not been favorably compared with experimental observations. For example, the expected large band gradient, a necessary condition for the intrinsic doping, has not been observed. Instead, the measured potential gradient was much smaller than expected.^[^ ^16^ ^]^ Moreover, the measured 2DEG density does not increase gradually with the LAO thickness, as predicted by the polar catastrophe model, but changes abruptly at the *t*_c_ and saturates thereafter.^[^ ^5^, ^6^ ^]^

Apart from the polar discontinuity, there is structural discontinuity across the LAO/STO heterointerface as well. While STO adopts an undistorted cubic phase, bulk LAO presents the antiferrodistortive (AFD) tilts of AlO_6_ octahedron at room temperature, which disturbs the continuity of the oxygen framework.^[^ ^17^ ^]^ For a subcritical LAO/STO heterostructure, it has been predicted that the internal field strongly polarizes the LAO lattice to produce a depolarization field.^[^ ^18^, ^19^, ^20^ ^]^ The AFD rotations, however, cannot mutually coexist with ferroelectric (FE) polar distortion in most perovskite oxides. This indicates that for a subcritical LAO/STO heterostructure the AFD rotations are likely to be suppressed. In this context, it is worth noting a recent study reporting the concurrent emergence of the two transitions in LAO/STO heterostructure at the *t*_c_; a metal‐insulator transition driven by the 2DEG formation in the STO side of interface and structural transition in the LAO side involving lattice distortions in which the FE polar state disappears and AFD modes emerge in their place.^[^ ^21^ ^]^

The fact that the two transitions occur simultaneously and are closely related to the polar field induced in the LAO suggests an important role of defects, especially, *V*_O_. Many DFT calculations already predicted that the formation energy of *V*_O_ at the surface of LAO/STO heterostructure decreases with LAO thickness and becomes zero when the *t*_c_ is reached.^[^ ^11^, ^13^, ^22^ ^]^ Moreover, the shallow donor level associated with *V*_O_ in LAO has a higher energy level than the bottom of the STO interface conduction band, so that the released electrons, driven by the internal polar field, can be transferred to the STO side of the interface.^[^ ^13^ ^]^ Recently, the *V*_O_ model is receiving increasingly positive feedback as a formation mechanism of 2DEG, since it is highly compatible with most of experimental observations. *V*_O_ is also known to induce local AFD rotations of BO_6_ octahedron in ABO_3_ perovskites.^[^ ^23^, ^24^ ^]^ Therefore, considering the shallow donor behavior and local symmetry breaking, *V*_O_ can relieve the electrical field by electron‐doping of the interface and induce the AFD octahedral rotations. While there have been some experimental studies reporting the oxygen deficiency of the surface of LAO/STO heterostructure,^[^ ^25^ ^]^ there was no direct observation revealing the formation of surface *V*_O_ and its correlation with the emergence of 2DEG and AFD.

Our comprehensive scanning transmission electron microscopy (STEM) analyses in conjunction with DFT calculations critically assess the LAO/STO heterostructures in light of the proposed models for the 2DEG formation and provide explicit STEM electron energy loss spectroscopy (EELS) evidence supporting the surface *V*_O_ model. The AFD octahedral rotations in LAO, which are suppressed in favor of FE distortion below the *t*_c_, evolves with the formation of surface *V*_O_ above the *t*_c_. The present study resolves a longstanding debate on the origin of 2DEG and unveils hitherto unknown correlation between structural and electronic transitions through *V*_O_ formation in the oxide heterostructure.

The formation of (charged) point defects can be considered at the boundaries of LAO/STO heterostructure, i.e., the LAO/STO interface and the LAO surface,^[^ ^12^, ^13^ ^]^ if they, together with accompanying free charge carriers, can cancel the polar field in the LAO film. The defect formation becomes energetically favored when the gain of electrostatic energy compensates for the energy cost of defect formation. Previously, interface defects have garnered considerable attention as a potential source of the 2DEG at LAO/STO interface as it was shown that cation intermixing occurs,^[^ ^7^, ^26^ ^]^ and that the LAO/STO interface is not as chemically abrupt as often assumed. Among the cation antisite defects between LAO and STO, depending on which cations undergo site exchange and their relative proportions, some of them can in principle cancel the polar field and also promote the interface conductivity via electron doping.^[^ ^8^ ^]^

The cation intermixing at the LAO/STO interface was examined by atomic‐resolution STEM energy dispersive X‐ray spectroscopy (EDS) elemental mapping. For both 3 and 10 u.c. samples, the EDS elemental maps and profiles revealed that A‐ and B‐site cations exchange (La ⇔ Sr and Ti ⇔ Al) occurred across the interface in nearly equal proportions (correlated substitution leading to La_1−_ *_x_* Sr*_x_* Al_1−_ *_y_* Ti*_y_* where *x* = *y*) (inset in **Figure** **1**a,c and Figure S1, Supporting Information). The maximal mixing appears in the first u.c. of each layer. After the first u.c. from the interface, the intermixing drops rapidly below 20% and the composition of each material becomes stoichiometric in the subsequent layers. The observed cation intermixing is in accord with the previous studies.^[^ ^8^, ^26^ ^]^ Given that the A‐ and B‐site exchange occurred, pairs of antisite defects (Ti_Al_ + Ai_Ti_ and La_Sr_ + Sr_La_) can form across the interface. These cation antisite defects have been studied extensively in light of the formation energy, induced dipole field, and the formation of free charge carriers.^[^ ^13^ ^]^ Further detailed analysis of STEM EDS results (Figure S2, Supporting Information) and discussion on the defect chemistry is given in the Supporting Information. Nonetheless, the fact that a similar extent of A‐ and B‐site exchange was observed in both insulating 3 u.c. and conducive 10 u.c. LAO/STO samples disproves the cation intermixing as a major source of the interface 2DEG.

**Figure 1:**
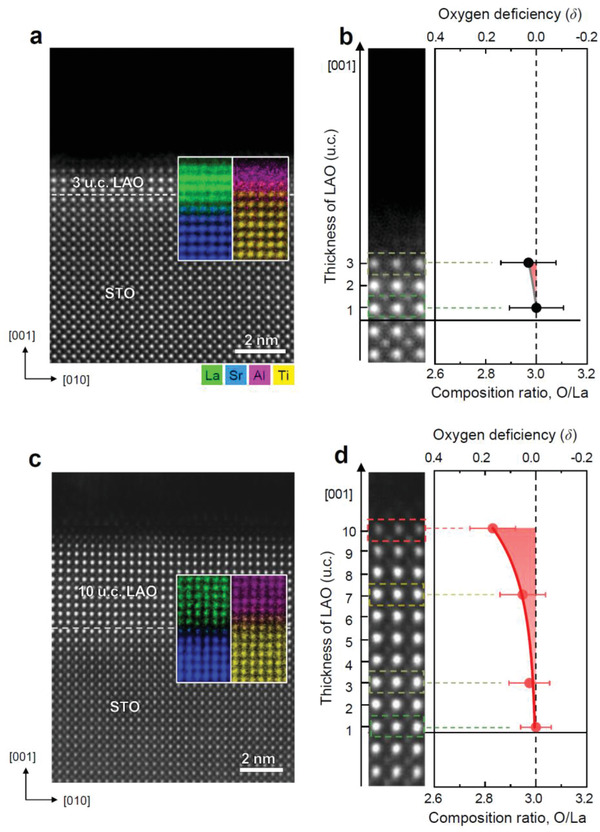
Interface and surface composition of LAO/STO heterostructures characterized by STEM EDS and EELS. a,c) Atomic‐column‐resolved STEMEDS elemental maps. STEMEDS elemental maps constructed by selecting La‐L_*α*_ and Al‐K_*α*_ for LAO and Sr‐L_*α*_ and Ti‐K_*α*_ for STO are overlaid on STEMHAADF image of 3 and 10 u.c. LAO/STO, respectively. b,d) O/La ratio plotted with the distance from LAO/STO interface of 3 and 10 u.c. LAO/STO, respectively. The O/La composition ratio was determined by areal integration of STEM EELS O‐K and La‐M_4,5_ edges. The STEM probe size for EELS measurements was 1.3 Å. The O/La ratio in the 10 u.c. LAO/STO starts deviating from about 7 u.c. and reaches 2.83 at the LAO surface. The oxygen deficiency (*δ*, LaAlO_3−_ *_*δ*_*) in 3 and 10 u.c. LAO/STO was measured to be 0.03 (LAO_2.97_) and 0.26 (LAO_2.74_). From the measured oxygen deficiency of 3 and 10 u.c. LAO/STO, the oxygen concentration is calculated to be 0.03*V*_O_/*a* ^2^ and 0.26*V*_O_/*a* ^2^ which corresponds to 1% and 8.7%, respectively.

The electric field maps obtained by inline electron holography show that the net field within the LAO of both 3 and 10 u.c. samples is small, indicating the compensation of the polar electric field independent of the film thickness (Figure S3, Supporting Information). However, there are local fields at the LAO surface and the LAO/STO interface with opposite signs, which are much stronger in the 10 u.c. sample. While the field at the LAO/STO interface is due to the negative charges corresponding to the 2DEGs, the field at the LAO surface indicates the presence of positive charges that are likely *V*_O_.^[^ ^27^, ^28^ ^]^

We analyzed a series of EELS O‐K edge obtained from the LAO/STO interface to the LAO surface of 3 and 10 u.c. samples. The oxygen composition was determined quantitatively by integrating the O‐K edge and normalizing it with that of the La‐M_4,5_ edge (Figure 1b,d). The O/La ratio measured for stoichiometric LAO is 3.0 but it decreases with the increase of *V*_O_ (if we assume that the La occupancy remains constant). Figure 1d shows that the O/La ratio in the 10 u.c. LAO/STO starts deviating from about 7 u.c. and reaches 2.83 at the LAO surface. To determine the *V*_O_ concentration, the oxygen deficiency (*δ* as in LAO_3−_ *_*δ*_*), i.e., deviation from the stoichiometric O/La ratio of 3.0, at each location of LAO from the interface to surface was integrated (represented by red shaded area in Figure 1b,d). The oxygen deficiency in 3 and 10 u.c. LAO/STO was measured to be 0.03 (LAO_2.97_) and 0.26 (LAO_2.74_), respectively. As one La is contained in one LAO unit cell area (*a* ^2^), the oxygen deficiency *δ* in the O/La ratio can be converted to the vacancy concertation in the unit of *V*_O_/*a* ^2^. From the measured oxygen deficiency of 3 and 10 u.c. LAO/STO, the oxygen concentration is calculated to be 0.03*V*_O_/*a* ^2^ and 0.26*V*_O_/*a* ^2^ which corresponds to 1% and 8.7%, respectively. Ideally, the amount of 2DEG required for charge compensation at the interface is ≈0.5e/*a* ^2^ (≈3.4 × 10^14^ cm^−2^). Assuming that each *V*_O_ generates two electrons, the required *V*_O_ concentration is ≈0.25*V*_O_/*a* ^2^ (≈1.7 × 10^14^ cm^−2^), which is far beyond the ordinary *V*_O_ concentration can be generated by thermal activation (e.g., ≈10^2^ cm^−2^ for the formation energy of ≈2.3 eV at 900 K) (Figure S4, Supporting Information).^[^ ^29^ ^]^ The measured *V*_O_ of 8.7% (0.26 *V*_O_/*a* ^2^) in the 10 u.c. LAO/STO agrees well with the ideal concentration of 8.3% (0.25e/*a* ^2^) which is required to compensate the polar electric field. In contrast, no measurable oxygen deficiency was not detected in the 3 u.c. LAO/STO (Figure 1b).

To check whether the oxygen deficiency detected in the 10 u.c. LAO/STO surface was induced during the TEM sample preparation, especially by the preferential thinning of the LAO surface region, we have carried out the control experiments using the cross‐sectional TEM specimens of single crystal LAO (001) substrate made by following the same TEM sample preparation method (Figure S5, Supporting Information). Although the TEM specimen of LAO single crystal exhibited a similar thickness variation toward the LAO (001) edge as in the LAO/STO samples, the O/La ratio remained constant and stoichiometric at 3.0. The fact that the single‐crystal LAO and the 3 u.c. LAO/STO (001) samples show the stoichiometric O/La ratio at the surface demonstrates that the oxygen deficiency detected near the 10 u.c. LAO/STO surface is due to the presence of *V*_O_ not due to the artifacts arising from TEM sample preparation.

To detect the changes made by *V*_O_ in the electron‐loss near‐edge structure (ELNES) of O‐K edge, the EELS O‐K edges from the LAO/STO interface to the LAO surface were examined in detail (**Figure** **2**). The EELS O‐K edge of LAO consists of the two major peaks with distinct fine structures. We notice that the shoulder peak (black arrow in Figure 2a,b) appearing at a lower energy of the first major peak is pronouncing at the first unit cell above the interface but becomes blurred at the surface of LAO film, e.g., 3 u.c. and 10 u.c. of the 3 u.c. and the 10 u.c LAO/STO sample, respectively. We further noticed that the overall fine structures of the surface O‐K edge of the 10 u.c. LAO/STO are blurred more, which is accompanied by an increase of the intensity at the valley between the two major peaks (dash lines in Figure 2b).

**Figure 2:**
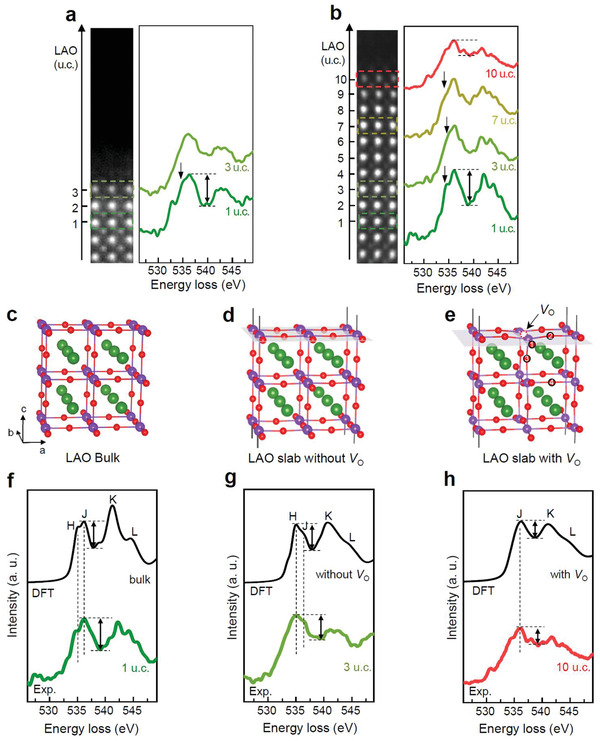
STEM EELS and DFT calculation of O‐K edge showing the formation of *V*_O_ at LAO/STO surface above *t*_c_. a,b) EELS O‐K edges obtained at various locations from the LAO/STO interface for 3 and 10 u.c. LAO/STO samples, respectively. c,d,e) Simulation cells for DFT calculation of EELS O‐K edge representing the oxygen atoms in: c) bulk LAO; d) LAO surface without *V*_O_; e) LAO surface with *V*_O_. One *V*_O_ was introduced to the surface AlO_2_ layer of the 2 × 2 × 2 supercell. In the LAO slab with *V*_O_, the EELS O‐K edge was calculated for oxygen at four different sites (indicated as 1–4) with respect to the vacancy position and various weighted averages were calculated to determine the best fit to the STEM EELS data (Figure S7, Supporting Information). A single electron core‐hole was added to all four O atoms. EELS O‐K edge calculated by DFT for the oxygen atoms in: f) bulk LAO; g) LAO surface without *V*_O_; h) LAO surface with *V*_O_. For comparison, the EELS O‐K edges obtained at 1 u.c. of the 10 u.c. sample, 3 u.c. of the 3 u.c. sample, and 10 u.c. of the 10 u.c. sample are added in (f), (g), and (h), respectively. The origin of fine structures labeled H, J, K, and L is explained in the text. The surface effect makes H‐J and K‐L peak separation blur but the intensity at the valley between the two major peaks remains the same as in the bulk (black arrow). While the surface effect suppresses the intensity of J peak but H peak remains unaffected, which results in asymmetry of the first major peak, the surface *V*_O_ results in the disappearance of H peak, resulting in a featureless single peak.

We carried out the DFT calculations of the O‐K edge for the oxygen with different atomic configurations in LAO. The EELS O‐K edge calculated for the oxygen within LAO bulk serves as the reference (Figure 2c,f). The peaks labeled H and J in the first major peak originate from the transition from O 1*s* to the hybridized O 2*p* and La 5*d* states, which are separated by ≈1.7 eV due to the *t*_2g_ *–e*_g_ crystal‐field splitting in the octahedral coordination.^[^ ^30^ ^]^ The peak labeled K originates from the transition to the hybridized O 2*p* and Al 3*p* states, and the peak L from the hybridized O 2*p* and La 6*sp* states.^[^ ^30^ ^]^ We note that the DFT calculation shown in Figure 2c,f has been performed on an ideal cubic perovskite structure without octahedral rotation. When the octahedral rotation (*a* ^−^ *a* ^−^ *a* ^−^) is considered, the DFT calculation yielded almost the same result, i.e., the H and J peaks are separated by ≈1.7 eV due to the *t*_2g_ *–e*_g_ crystal‐field splitting (Figure S6, Supporting Information).

The EELS O‐K edge calculated for the surface oxygen of AlO_2_‐terminated LAO slab (Figure 2d) shows that the surface effect makes noticeable changes in the peak separation due to the incomplete octahedron at the surface; H‐J and K‐L peak separation are blurred (Figure 2g). In specific, the surface effect suppresses the intensity of peak J but peak H remains unaffected, resulting in an asymmetric shape of the first major peak. However, the intensity at the valley between the two major peaks remains the same as in the bulk LAO (black arrow). The EELS O‐K edge obtained from the surface of 3 u.c. LAO is compared very well with the DFT calculation (Figure 2g).

The DFT calculation of EELS O‐K edge was performed by removing one oxygen atom from the AlO_2_‐terminated surface to investigate the effects made by *V*_O_ (Figure 2e). We note that the calculated EELS O‐K edge from the LAO slab (Figure 2h) is a weighted average of the O‐K edge of the four nearest oxygen atoms to the *V*_O_ (indicated as 1, 2, 3, 4 in Figure 2h and Figure S7, Supporting Information). There are two major changes in the EELS O‐K edge due to the presence of surface *V*_O_, which are: the doublets of the two peaks (peaks H and J, peaks K and L) merge into a single peak, and the deep gap between peaks J and K becomes a shallow gap. The merged broad peaks in the LAO slab suggest that *V*_O_ breaks the octahedral symmetry by changing the relative distance between La and O atoms, and then modifies the hybridization between La 5*d* and O 2*p* states. Moreover, the *V*_O_ usually reduces the local symmetry by introducing lattice distortion, which generates a broad distribution of localized states, resulting in the shallow gap. These significant changes in the EELS O‐K edge induced by *V*_O_ can also be recognized by referring to the projected density of states of O 2*p* of the LAO slab (Figure S8, Supporting Information). The merged broad peaks of EELS O‐K edge with a shallow gap obtained from the calculated O‐K edge with *V*_O_ agrees well with the surface O‐K edge of 10 u.c. LAO (Figure 2h and Figure S9, Supporting Information). We note that these features are certainly different from the changes made by the surface effects without *V*_O_, i.e., the asymmetric first major peak due to H‐J peak splitting and the deep gap between the two major peaks (Figure 2g). Therefore, as confirmed by both DFT calculations and experimental EELS measurements, the noticeable modification of the EELS O‐K edge fine structure originates from a nonnegligible density of *V*_O_ present at the LAO surface.

We checked the possible formation of *V*_O_ in the STO side of the interface as well, which can also act as a source of the 2DEG. There exist the Ti atoms with Ti^3+^ state near the interface due to the occupation of 2DEG (Figure S10, Supporting Information) but this cannot be used to conclude the presence of *V*_O_. The O‐K edge line profile simultaneously obtained with Ti‐L_2,3_ edge was analyzed to determine whether the Ti^3+^ state originates from *V*_O_ in STO or not. It is known that *V*_O_ in STO can be detected by analyzing the fine structure of EELS O‐K edge, particularly, the intensity ratio of the first two peaks.^[^ ^20^ ^]^ As presented in Figure S10 in the Supporting Information, the intensity ratio indicates the absence of *V*_O_ in the STO substrate.

**Figure** **3**a,b show STEMhigh‐angle annular dark‐field (HAADF) images and the out‐of‐plane lattice constants measured by determining the center‐of‐mass of the atomic column intensities, respectively. The out‐of‐plane lattice constants were measured separately from the A‐site and the B‐site cations to trace any difference in the strain and relaxation behavior of each sub‐lattice. The results from both 3 and 10 u.c. samples showed the lattice expansion of STO near the LAO/STO interface, which is a well‐known phenomenon already observed by X‐ray diffraction.^[^ ^7^, ^9^, ^19^, ^31^ ^]^ The origin of the lattice expansion has been attributed to: the electrostrictive effect,^[^ ^31^ ^]^ the change of valence state from Ti^4+^ to Ti^3+^,^[^ ^7^, ^9^ ^]^ the dilatory distortion resulting from chemical intermixing effects,^[^ ^19^ ^]^ and the lattice expansion induced by Sr vacancies, *V*_Sr_^[^ ^32^, ^33^ ^]^ or *V*_O_.^[^ ^33^ ^]^ The lattice expansion is not attributable to the change of valence of Ti since the 3 u.c. LAO/STO without 2DEG also exhibits a similar behavior. As no *V*_O_ is detected near the STO substrate by EELS, the expansion is not likely caused by *V*_O_ (Figure S10, Supporting Information). Considering that the similar lattice expansion was observed for both 3 and 10 u.c. samples, the lattice expansion is likely induced by the cation intermixing involving the formation of antisite defects. We note that DFT calculations in the present study were performed in order to provide the benchmark results of a perfect interface in the absence of cation intermixing. Such interface intermixing would be very difficult to investigate with DFT because of the unrealistic computational effort. Since Pauli et al.^[^ ^19^ ^]^ previously considered the influence of intermixing with DFT and found that the differences between the abrupt‐ and intermixed DFT models are so small, it is not expected that considering intermixing over a larger depth will have a significant effect on the relaxation of surface atoms with or without *V*_O_ at the LAO surface.

**Figure 3:**
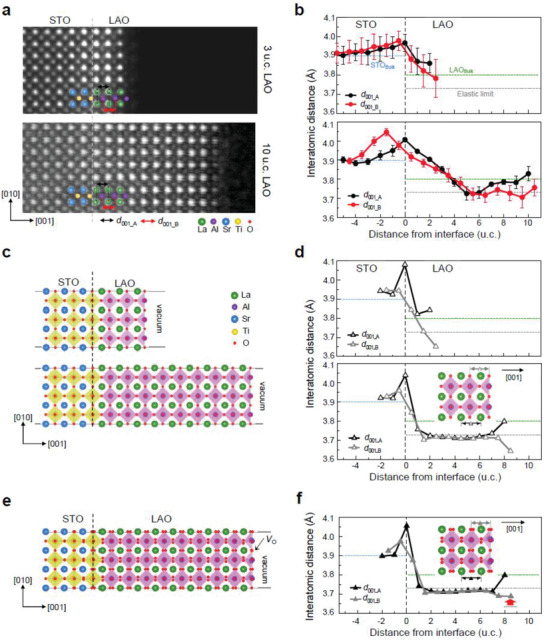
Lattice constants of LAO/STO heterostructures measured by STEMHAADF and DFT. a) STEMHAADF images taken at the [100] zone axis of 3 u.c. (upper) and 10 u.c. (lower) samples. b) Plot of the A‐site lattice constant (*d*_001_A_) and B‐site lattice constant (*d*_001_B_) along the [001] out‐of‐plane direction for 3 u.c. (upper) and the 10 u.c. (lower) samples. Each data point is an average over 30 u.c. along the [010] in‐plane direction. The error bars represent the standard deviation. c) Atomic models (relaxed) of 3 u.c. (upper) and 10 u.c. (lower) LAO/STO heterostructures without surface *V*_O_after DFT calculation. d) Plot of the A‐site lattice constant (*d*_001_A_) and B‐site lattice constant (*d*_001_B_) along the [001] out‐of‐plane direction for 3 u.c. (upper) and the 10 u.c. (lower) samples. e) Atomic models (relaxed) of 9 u.c. LAO/STO heterostructures with surface *V*_O_after DFT calculation. d) Plot of the A‐site lattice constant (*d*_001_A_) and B‐site lattice constant (*d*_001_B_) along the [001] out‐of‐plane direction of 9 u.c. LAO/STO with surface *V*_O_. The red arrow in (f) highlights a different lattice relaxation behavior of the A‐site cations at surface in the presence of *V*_O_, which matches well with the experimental measurement in (b).

The LAO lattice in an LAO/STO heterostructure is tensile strained along the in‐plane direction to match the STO lattice (Figure S11, Supporting Information). Due to the Poisson effect, the out‐of‐plane lattice parameter of LAO decreases below the bulk value accordinlgy. However, the lattice constants decrease gradually over 3–5 u.c. from the interface, which can be attributed to the electrostrictive effects and/or cation intermixing. Cacellieri et al.^[^ ^31^ ^]^ have shown that a small electric field remained in LAO film can cause the expansion of LAO lattice, which increases quadratically with the field. Pauli et al.^[^ ^19^ ^]^ have shown that the cation intermixing extends over 3 u.c., which causes the extended variation of lattice constant across the interface. All these measurements, including another shown in Figure S12b in the Supporting Information, are in good agreement with our results.

In addition to the epitaxial strain, the LAO surface exhibits unique surface relaxation. One should note that the LAO (100) surface is a polar surface and thus susceptible to the structural and/or chemical reconstruction to compensate the surface charges, such as: rumpling of the surface atoms, adatom absorption, vacancy formation, and electronic reconstruction involving screening charge accumulation.^[^ ^34^ ^]^ An additional degree of complexity may arise in the LAO (001) surface when this polar oxide is in direct contact with nonpolar STO as the polar field is induced and grows with the film thickness. In the DFT calculation without surface *V*_O_, the La atoms in the sub‐surface LaO layer are displaced toward the surface, leading to the expansion of the A‐site cation lattice constant, but in the surface AlO_2_ layer both Al and O atoms move toward the interface by similar amounts,^[^ ^18^ ^]^ resulting in the contraction of the B‐site cation lattice constant (Figure 3c,d). The DFT calculation agrees very well with the literature^[^ ^18^, ^19^, ^33^ ^]^ (Figure S12c–e, Supporting Information) and also with the experimental measurement from the 3 u.c. LAO/STO sample (Figure 3b). We note that this kind of characteristic surface relaxation, i.e., the contraction of the first surface unit cell, was also observed from a single‐crystal LAO (001) surface without *V*_O_ even though the surface termination (LaO^+^) of the single crystal is different from that (AlO_2_^−^) of LAO/STO (001) heterostructure (Figure S5d, Supporting Information).

After confirming the surface relaxation behavior of LAO (001) in the absence of *V*_O_, we carried out DFT calculation of 10 u.c. LAO/STO with or without *V*_O_ at the LAO surface. In the 10 u.c. LAO/STO cell without *V*_O_, the displacement of near surface atoms remained almost the same as in the 3 u.c. LAO/STO cell, leading to the expansion of the A‐site cation but the contraction of the B‐site cation lattice constants (Figure 3c,d). In the *V*_O_ model, however, the surface AlO_2_ layer shows different relaxation; the Al ions adjacent to the *V*_O_ are displaced upward due to the electrostatic repulsion from the positively charged *V*_O_,^[^ ^22^ ^]^ reducing the lattice contraction (Figure 3e,f). The experimental profile of the B‐site cation lattice constant of 10 u.c. LAO matches well with the DFT model with the *V*_O_, further supporting the formation of *V*_O_ at the LAO surface.

Apart from the surface relaxation, the FE distortion of LAO lattice can arise as consequence of a depolarization effect, which produces counter dipole moments that reduce the polar field. The FE distortion results in the buckling of O‐cation‐O chains such that the cations in the LaO and AlO_2_ layers are displaced toward the surface relative to the O atoms which is defined as positive depolarization buckling. Overall, the DFT calculation predicts that the polar electric field strongly polarizes the subcritical LAO lattice and the buckling of O‐La‐O chains is more pronounced than that of O‐Al‐O chains.^[^ ^18^, ^19^, ^22^ ^]^ In the 3 u.c. LAO film, a positive buckling is seen to exist together with the interface intermixing; both Al and O atoms move toward the interface with respect to La, however, the displacement of O atoms is much larger than displacement of Al atoms, resulting in the buckling of O‐Al‐O chains (**Figure** **4**), which is in qualitative agreement with the surface X‐ray diffraction study by Pauli et al.^[^ ^19^ ^]^ as well as with our DFT calculation. The results demonstrate that at sub‐critical thicknesses the built‐in polar field induced in the LAO film due to the charged atomic layers is compensated predominantly by FE distortion, which generates a depolarization field (Figure 4 and Figure S13a, Supporting Information).^[^ ^21^ ^]^ It is likely that the lattice distortion caused by the different ionic radii of intermixing cations can also promote the depolarization buckling of LAO or vice versa. Although the displacement of Ti and O atoms in the intermixed STO region below the interface is small in average, a distinct negative buckling is observed, which can also contribute to the screening of polar LAO/STO interface (Figure 4b,d). On the other hand, the FE distortion measured in the 10 u.c. LAO film was marginal (**Figure** **5**d and Figure S13b, Supporting Information), and is consistent with the compensation of the polar field predominantly by 2DEG formation.

**Figure 4:**
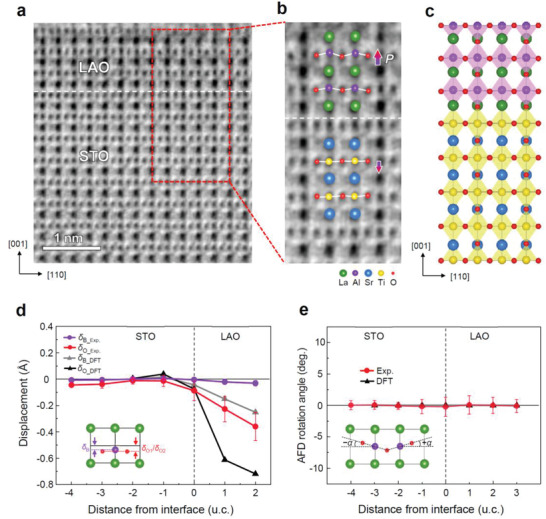
FE polar distortion and AFD rotation of 3 u.c. LAO/STO. a) STEM ABF image taken along the $\left[1\bar{1}0\right]$ zone axis. b) Enlarged view of the selected region (marked as red dashed rectangle in (a)), showing FE polar distortion with superimposed atomic model. c) Fully relaxed 3 u.c. LAO/STO heterostructure by DFT calculation. d) Plot of the polar displacements of B‐site cations (*δ*_B_) and O atoms (*δ*_O_ = *δ*_O1_+*δ*_O2_) measured from STEM ABF image and DFT calculation. The displacement of B‐site cations (*δ*_B_) and O atoms (*δ*_O1,_ *δ*_O2_) was determined as the distance (*δ*) from the center‐of‐mass of A‐site cation lattice (refer to inset). e) Plot of the AFD rotation angle measured from the STEM ABF image and DFT calculation. The AFD rotation angle is defined as a projected O‐Al‐O tilt angle *α* corresponding to the amplitude of the ripple (refer to inset). Each data point of AFD rotation angle and FE polar displacement is an average over 15 u.c. along the [110] in‐plane direction. The error bars represent the standard deviation. The dashed line marks the nominal interface.

**Figure 5:**
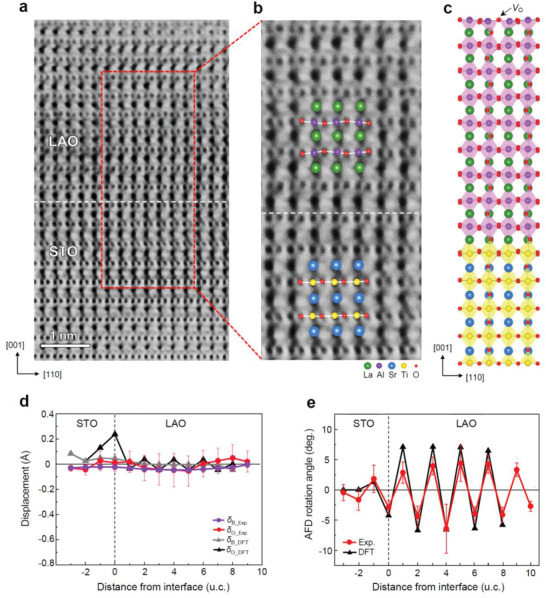
FE polar distortion and AFD rotation in 10 u.c. LAO/STO. a) STEM ABF image taken along the $\left[1\bar{1}0\right]$ zone axis. b) Enlarged view of the selected region (marked as red dashed rectangle in (a)), showing the AFD octahedral rotation with superimposed atomic model. c) Fully relaxed structure by DFT calculation for 9 u.c. LAO/STO cell with *V*_O_. d) Plot of the FE polar displacements of B‐site cations (*δ*_B_) and O atoms (*δ*_O_ = *δ*_O1_+*δ*_O2_) measured from STEM ABF image and DFT calculation. The large displacement of O atoms observed in the DFT result is caused by the occupation of 2DEG at LAO/STO interface, which can be confirmed by comparing the result with one that calculated without surface *V*_O_ (Figure S15, Supporting Information). e) Plot of the AFD rotation angle measured from the STEM ABF image and DFT calculation result of the *V*_O_ model. Each data point of AFD rotation angle and FE polar displacement is an average over 15 u.c. along the [110] in‐plane direction. The error bars represent the standard deviation. The dashed line marks the nominal interface. There exists the shift of atomic columns in the ABF image of STO substrate region due to the slight sample tilt, which degrades the reliability of measurement results in the STO substrate.

The bulk crystal structure of LAO deviates from the ideal $Pm\bar{3}m$ perovskite by out‐of‐phase rotations of AlO_6_ octahedra around the crystallographic [111] axis (*ϕ_abc_*, Glazer system *a* ^−^ *a* ^−^ *a* ^−^) that lower the symmetry to space group $R\bar{3}c$. According to Hatt and Spaldin,^[^ ^17^ ^]^ several different phases of LAO, characterized by distinct patterns of AFD octahedral rotations, can be stabilized by varying the epitaxial constraints. For example, the biaxial tensile strain (+2.97%) acting on the LAO in the LAO/STO heterostructure is predicted to stabilize the *Imma* phase, comprised of *ϕ_ab_* rotations (*a* ^−^ *a* ^−^ *c* ^0^) with a rotation axis along the in‐plane [110]_pc_ direction (“pc” stands for pseudo‐cubic). As the strain relaxation of LAO film is known to take place when the thickness is larger than 20 u.c.,^[^ ^31^ ^]^ both 3 and 10 u.c. LAO films are fully strained on STO (001) substrate with maintaining the same in‐plane lattice constant as STO. In the present study, the in‐plane lattice constants measured from STEM images also confirmed that both LAO films are fully strained on the STO substrate (Figure S11, Supporting Information). Therefore, the strain effect alone will favor the AFD rotation with the rotation axis along the in‐plane [110]_pc_, resulting in *a* ^−^ *a* ^−^ *c* ^0^ pattern without FE distortions.

However, it appears that the evolution of AFD rotation in an LAO/STO heterostructure is governed more strongly by the electric field than the mechanical strain—an uncompensated polar field in the LAO/STO heterostructure suppresses the AFD rotation in favor of FE displacements which produce a depolarization field that compensates the polar field. The AFD rotation can appear in LAO/STO heterostructure only when the polar field is compensated by the formation of 2DEG above the *t*_c_. Recently, Gazquez et al.^[^ ^21^ ^]^ found an LAO thickness‐dependent crossover between FE distortion and AFD rotations in LAO/STO heterostructures, indicating that there exists a competition between FE displacements and AFD rotations. For a subcritical LAO film (*t* < 4 u.c.), the FE distortions are dominated by the upward movement of La atoms, whereas for a thicker LAO film (*t* > 4 u.c.) the FE distortions are suppressed in favor of strong AFD rotations of the AlO_6_ octahedral networks. As such, the structural evolution of LAO/STO (001) heterostructures goes in line with the general notion that the nonpolar AFD rotation and polar FE distortion tend to compete and suppress each other in ABO_3_ perovskite oxides. As the evolution of AFD rotations in an LAO/STO heterostructure is closely related to the compensation of the polar field, which is in turn linked to the formation of surface *V*_O_.

To evaluate the AFD rotations by experiment, the LAO/STO heterostructures were investigated along the ${\left[1\bar{1}0\right]}_{\mathrm{pc}}$ zone axis in STEM annular bright‐field (ABF) mode. In the 3 u.c. LAO/STO, AFD rotations are completely suppressed in favor of a strong FE distortion within the film (Figure 4d,e). In the 10 u.c. LAO film, however, the opposite behavior was observed; pronounced AFD rotations were observed while the FE distortions were suppressed (Figure 5d,e). The rotation angles are overall quite large (*α* ≈ ±3°–7°) within the LAO film and progressively decrease to zero toward the STO substrate (Figure 5e). Both the measured in‐plane tilt axis and the rotation angle are consistent with our DFT calculations and also with the first‐principle predictions for tensile strained (field‐free) LAO films by Hatt and Spaldin.^[^ ^17^ ^]^ The AFD rotation penetrates into the STO about 2 u.c. below the interface and decreases to zero thereafter, which agrees well with the previous report by Jia et al.^[^ ^35^ ^]^

In order to verify whether the surface *V*_O_ indeed triggers the AFD rotations, one oxygen is removed from the AlO_2_‐terminated LAO surface of the LAO/STO heterostructure in the DFT calculation (Figure S14, Supporting Information), and then the structure is fully relaxed until the forces are less than 0.5 × 10^−2^ eV Å^−1^. As a reference, the control DFT calculation performed with a *V*_O_‐free LAO/STO slab shows that even the small internal field remained in the 9 u.c. LAO effectively suppresses AFD rotation, whereas it induces FE distortion which produces a depolarization field (Figure S15, Supporting Information). When the *V*_O_ is introduced to the LAO surface, we find that noticeable AFD rotations are apparently induced across the entire LAO region. The AFD rotations of the AlO_6_ octahedra are in opposite directions in adjacent cells and seen as a ripple pattern of Al‐O‐Al chains along the [110]_pc_ direction (Figure 5b). We quantify the AFD rotation as a rotation angle *α* projected along ${\left[1\bar{1}0\right]}_{\mathrm{pc}}$ [110]_pc_ direction, corresponding to the amplitude of the ripple, which is measured as *α* ≈ ±7° from the DFT calculation (Figure 5e). In particular, the polar field inside LAO is indeed completely cancelled by the single *V*_O_ per 2 × 2 LAO unit cell of the surface (Figure S14, Supporting Information). This result clearly shows that the emergence of AFD rotations in the LAO/STO heterostructure is critically controlled by the internal fields in LAO and thus by *V*_O_ formation at the LAO surface; this is because the surface *V*_O_ can cancel the internal field by electron doping and promote structural relaxation of the surface Al‐O‐Al chains.

We note that the metallic conductivity arising at an interface between two insulating oxides may have diverse origins. The mechanism proposed in this study, i.e., the polar field‐induced *V*_O_ formation at the surface of crystalline LAO/STO heterostructure, is just one mechanism that works for a specific polar/nonpolar oxide combination and when this heterostructure is grown at ordinary epitaxial growth conditions, e.g., high temperature and oxygen‐rich environment. There exists, however, different origin of 2DEG, which is mainly driven by oxygen outward diffusion from a substrate and associated *V*_O_ formation and/or concomitant chemical redox reactions of plasma species at the oxide interface resulting in the formation of 2DEG.^[^ ^36^, ^37^, ^38^ ^]^ This mechanism is preferred especially for oxygen‐deficient LAO overlayer grown at room temperature and/or under the impingement of high energetic plasma species driving chemical redox reaction reducing STO substrate.^[^ ^36^, ^37^ ^]^ In addition, a similar mechanism has been suggested to work for the high mobility 2DEG at (001)‐oriented *γ*‐Al_2_O_3_/STO spinel/perovskite heterointerface^[^ ^38^ ^]^ where each layer is nominally charge neutral, so that polar field‐induced *V*_O_ formation as expected in the standard (crystalline) LAO/STO interface may not contribute. For an LAO/STO heterostructure, if surface redox reaction involving *V*_O_ formation at an STO substrate is suppressed by growing LAO film at high growth temperature and oxygen partial pressure, then the polar instability growing with the growth of LAO film will activate the formation of *V*_O_ at the LAO surface as we observed in the present study.

Comprehensive STEM analyses of the LAO/STO heterostructures demonstrate that surface *V*_O_ is the most probable source of the interface conductivity. While cation intermixing occurs spontaneously and independent of the film thickness, *V*_O_ forms at the LAO surface only when the film thickness exceeds the *t*_c_ for 2DEG formation. Below the *t*_c_, the polar field in LAO is compensated primarily by the depolarization fields generated by the FE distortions. With the formation of surface *V*_O_, the FE distortions of LAO disappear and the bulk‐like AFD rotation is stabilized. The present study directly shows the critical roles of *V*_O_ not only in the formation of 2DEGs but also in the competitive evolution of FE distortion and AFD rotation in oxide heterostructures; further, it emphasizes the importance of the assessment of multiple aspects of interface information for the correct understanding of the existence of correlated transitions and the emergent interface properties of oxide heterostructures. Our work resolves a longstanding debate on the origin of 2DEG in oxide heterostructures and paves the way for defect engineering of oxide electronics.

## Experimental Section

### Materials System

The LAO/STO heterostructures were produced by epitaxial growth of an LAO film on STO substrate by using pulsed‐laser‐deposition. The growth temperature and oxygen partial pressure was 550 °C and 10^−3^ mbar, respectively. Before deposition, both STO (001) substrates were chemically etched with buffered hydrofluoric acid for 60 s and then annealed at 1000 °C for 6 h under oxygen flow to make single termination of TiO_2_ for the (001) surface with controlled step and terrace structures. The epitaxial LAO films were grown with thicknesses of 3 and 10 u.c. on an STO (001) substrate, where the thickness of 1 u.c. assuming a pseudocubic unit cell of LAO, was 3.8 Å. During the growth of the LAO films, reflection high energy electron diffraction (RHEED) intensity oscillations were obtained in‐situ with the electron beam being aligned parallel to the [001] of STO (001) substrate. The periodic RHEED oscillations verified that the growth occurred in a layer‐by‐layer manner. Atomic force microscopy surface topology images of the grown samples revealed a well‐defined step‐terrace structure for both samples.

### Inline Electron Holography

Cross‐sectional TEM samples for inline electron holography were prepared by mechanical grinding to a thickness of around 80 µm and dimpling to a thickness of less than 10 µm. The dimpled samples were then ion‐milled using first a 3 kV Ar^+^ ion beam (PIPS, Gatan, Inc.) and then a low energy (0.5 kV) Ar^+^ ion beam to remove surface damage layers. Inline electron holography was carried out using a field‐emission TEM (JEM‐2100F, JEOL) operated at 200 kV. An objective aperture of 10 µm in diameter was used to select the transmitted beam, which limited the spatial resolution to 0.8 nm. Bright‐field (BF) TEM images at defocus values ranging from −4 to +4 µm were acquired in 1 µm step by exposing 2048 × 2048 pixels fiber‐optically coupled camera (UltraScan 1000 FT, Gatan, Inc.) for 4 s. All images were recorded using the Gatan's GIF Tridiem imaging filter to remove inelastically scattered electrons outside an energy window of 0 ± 7.5 eV. In order to minimize electron beam damage and also to secure a large field of view, the BF TEM images were obtained at a low magnification under low electron dose conditions. The obtained inline electron holograms were used to reconstruct the phase shift of the transmitted beam by using the full resolution wave reconstruction (FRWR) algorithm.^[^ ^39^ ^]^ In order to extract the correct electric field, the local sample thickness was calibrated using EELS log‐ratio technique. The component *E_y_* of the electric field vector **E** = (*E_x_*,*E_y_*) along the interface normal was determined using the thickness‐calibrated potential profile (*V_y_*) according to *E_y_* = −∇*V_y_*.

### STEM Imaging, EELS, and EDS

STEMHAADF and ABF imaging and EELS were performed by using a JEM‐2100F (Jeol Ltd., Japan) equipped with a spherical aberration corrector (CEOS GmbH). The probe convergence angle of ≈22 mrad was used. The inner and outer angles of the HAADF detectors were 90 and 200 mrad, respectively. The obtained STEMHAADF and ABF images were band‐pass filtered to reduce background noise (HREM Research Inc.). The electron dose for STEM imaging was about 1.1 × 10^6^ e nm^−2^ s^−1^. EEL spectra and spectrum images were obtained at 200 kV using an EEL spectrometer (Gatan GIF Quantum ER, USA) with an energy resolution of 0.8 eV. To evaluate how the electron probe introduces defects or modifies the charge balance, the threshold electron dose rate was determined above which a detectable amount of damages was produced by monitoring the white line intensity ratios and the fine structures of EELS Ti‐L_2,3_ edge of STO and La‐M_4,5_ edge of LAO during the acquisition. The electron dose rate used for STEM EELS was 4.0 × 10^7^ e nm^−2^ s^−1^ which was below the measured threshold (5.7 × 10^7^ e nm^−2^ s^−1^). The atomic‐resolution STEM EDS chemical mapping was carried out on a JEM‐ARM 200F (Jeol Ltd., Japan) equipped with a spherical aberration corrector (ASCOR, CEOS) and energy dispersive X‐ray spectrometer (JED‐EDS, JEOL). A fast atomic‐scale EDS mapping data of the sample within several ten minutes was acquired by utilizing dual‐type EDS detector (the effective X‐ray detection area of a 100 mm^2^ for each) with a large effective solid angle (≈0.8 sr) and a highly focused electron probe (≈1.1 Å) at the electron dose rate of 7.7 × 10^6^ e nm^−2^ s^−1^. The resulting elemental maps were obtained by the multiple frame summation up to less than 500 frames with 256 × 256 pixels resolution and the acquisition time of 10 µs per pixel (≈5.5 min in maximum as a total acquisition time). Background noise floor in each map was removed by applying a weak Wiener filter.

### EELS Calculation

Calculations using the CASTEP code^[^ ^40^ ^]^ were performed to simulate the O‐K ELNES edge for LAO. For the bulk LAO, 2 × 2 × 2 supercell was used, and the core‐hole was considered. In order to investigate the influence of surface *V*_O_ on the O‐K ELNES, one oxygen atom was removed from the AlO_2_ surface layer of 50 atoms‐based 2 × 2 × 2 LAO slab, and the single electron core‐hole was put in every four neighboring oxygen atoms around the surface *V*_O_. Depending on the distance from the *V*_O_, unequal weights of 0.6, 0.3, 0.1, and 0 were assigned to the EELS O‐K edges of four oxygen atoms (indicated in 1, 2, 3, and 4), respectively. A DFT calculation was carried out on EELS O‐K edge for 2 × 2 × 2 LAO slab without surface *V*_O_ to distinguish the surface effects from the *V*_O_ effects on the spectral features of O‐K edge. The plane wave basis set and on‐the‐fly generation ultrasoft pseudopotential were employed to describe exchange correlation function within the generalized gradient approximation‐Perdew–Burke–Ernzerhof (GGA‐PBE).^[^ ^41^ ^]^ The energy cutoff of 500 eV and 4 × 4 × 4 Monkhorst‐Pack grid of *k*‐points for all calculations were used, and core hole on a single oxygen atom was introduced to obtain intensity distribution of EEL spectra within the single particle approximation.

### Theoretical Modeling

The first‐principles DFT calculations were performed using the GGA‐PBE^[^ ^41^ ^]^ and the projector‐augmented wave method with a plane‐wave basis^[^ ^42^ ^]^ as implemented in the Vienna ab initio simulation package (VASP) code.^[^ ^43^ ^]^ For the Brillouin zone integration, a kinetic energy cut‐off of 450 eV and *Γ*‐centered 4 × 1 *k*‐point meshes for 2 × 2 in‐plane (LAO)_9_/(STO)_4_ (*n*‐type interface) slabs containing 272 atoms with a vacuum thickness of 16 Å were used. The in‐plane lattice constant of LAO/STO slabs was fixed to 2 × 2 *a*_STO_ obtained with PBE potential. The calculations were fully converged in energy to 10^−5^ eV per cell, and the structures were relaxed until the forces were less than 5 × 10^−2^ eV Å^−1^. The creation of oxygen vacancies was marked by removal of oxygen atoms at the AlO_2_‐terminated LAO surface. One oxygen vacancy was introduced on the 2 × 2 × 1 surface unit cells of 9 u.c. LAO/STO heterostructure, i.e., one oxygen vacancy per four unit cell (0.25*V*_O_/*a* ^2^) (≈1.7 × 10^14^ cm^−2^) to completely compensate the internal polar field. Assuming that an oxygen vacancy generated two electrons, this single oxygen vacancy could generate the ideal 2DEG density of 0.5e/*a* ^2^. The concentration of oxygen vacancy corresponded to 8.3% (one vacancy in total 12 oxygen atoms).

### Data Analysis for Atomic Displacement and Polarization

All of the STEMHAADF images were 2D Wiener filter to remove background noise (HREM Research Inc.). The position of each atomic column in STEMHAADF and ABF images was extracted by using the peak pairs analysis (HREM Research Inc.). From STEM ABF image, the atomic displacements of B‐site cations and O atoms from along the [001] direction were measured by distance *δ*_B_ and *δ*_O_ with respect to the positions of center of mass (COM) of A‐site cations. Note that it was estimated under the assumption that there were no displacement of O atom positions belonging to the AO planes. In DFT result, atomic displacements of B‐site cations were measured by distance *δ*_B_ with respect to the positions of COM of the A‐site cations. The displacements of O atoms were determined by shift of averaged position of O atoms belonging to the BO_2_ plane with respect to COM of A‐site cations. By considering the atomic displacement, polarization could be simply calculated by the following equation, $P=\frac{1}{V}\sum \delta_{i}Z_{i}$,^[^ ^44^ ^]^ where *V* is unit cell volume, *δ_i_* is the displacement of *i* atom from COM position, and *Z_i_* is the Born effective charge of *i* atom. The Born effective charges for polarization used reported value in literature.^[^ ^45^ ^]^

## Conflict of Interest

The authors declare no conflict of interest.

## Supporting information

Supporting Information

## Data Availability

The data that support the findings of this study are available from the corresponding author upon reasonable request.
