# Supplementary material for: Electronic and Structural Transitions of LaAlO3/SrTiO3 Heterostructure Driven by Polar Field‐Assisted Oxygen Vacancy Formation at the Surface
Source: Adv Sci (Weinh). 2021 May 24;8(14):2002073. doi: 10.1002/advs.202002073 (PMC8292910; doi:10.1002/advs.202002073)
Supplement: Supplementary file 1 — Supporting Information [file ADVS-8-2002073-s001.pdf]

© 2021 Wiley-VCH GmbH

Supporting Information

for *Adv. Sci.*, DOI: 10.1002/advs.202002073

Electronic and Structural Transitions of $LaAlO_3/SrTiO_3$ Heterostructure Driven by Polar Field Assisted Oxygen Vacancy Formation at the Surface

*Kyung Song, Taewon Min, Jinsol Seo, Sangwoo Ryu, Hyungwoo Lee, Zhipeng Wang, Si-Young Choi, Jaekwang Lee, Chang-Beom Eom, and Sang Ho Oh**

Supporting Information

Electronic and Structural Transitions of $LaAlO_3/SrTiO_3$ Heterostructure Driven by Polar Field Assisted Oxygen Vacancy Formation at the Surface

Kyung Song[1,2], *Taewon Min*[3], *Jinsol Seo*[4], *Sangwoo Ryu*[5], *Hyungwoo Lee*[5], *Zhipeng Wang*[4], *Si-Young Choi*[2,1], *Jaekwang Lee*[3], *Chang-Beom Eom*[5], *and Sang Ho Oh*[1,4]*

[1]Department of Materials Science and Engineering, Pohang University of Science and Technology (POSTECH), Pohang 37673, Republic of Korea

[2] Materials Testing and Reliability Division, Korea Institute of Materials Science (KIMS), Changwon 51508, Republic of Korea

[3]Department of Physics, Pusan National University, Busan 46241, Republic of Korea

[4]Department of Energy Science, Sungkyunkwan University, Suwon 16419, Republic of Korea

[5]Department of Materials Science and Engineering, University of Wisconsin-Madison, Madison, Wisconsin 53706, USA

*e-mail: sanghooh@skku.edu

Discussion of cation intermixing model of 2DEG formation based on STEM EDS results

For a pair of anti-site defects to compensate the polar field across the LAO/STO interface, the dipole field generated by the defects should point in the opposite direction to the polar field.[1] For example, the $\mathrm{Ti}_{\mathrm{Al}}^{\bullet}+\mathrm{Al}_{\mathrm{Ti}}'$ anti-site defect pair formed by Al ⇔ Ti site exchange induces the dipole field, which is opposite to the polar field, so that their formation is favored. However, $\mathrm{Al}_{\mathrm{Ti}}'$ is a deep level defect that traps the electron generated by $\mathrm{Ti}_{\mathrm{Al}}^{\bullet}$.[2] As such, the $\mathrm{Ti}_{\mathrm{Al}}^{\bullet}+\mathrm{Al}_{\mathrm{Ti}}'$ anti-site defect can reduce the polar field but cannot generate the interface conductivity. In the case of $\mathrm{Sr}_{\mathrm{La}}' + \mathrm{La}_{\mathrm{Sr}}^{\bullet}$ anti-site defects, the dipole field points in the same direction as the polar field, so their formation is not favored. However, La ⇔ Sr site exchange can occur when it is accompanied by Al ⇔ Ti site exchange.[2] Although La can act as donor in STO and thus can release charge carriers, the formation of itinerant electrons by the donor defect $\mathrm{La}_{\mathrm{Sr}}^{\bullet}$ is, however, still controversial. Nonetheless, the fact that a similar extent of La ⇔ Sr site exchange was also observed in the insulating 3 u.c. LAO/STO sample disproves the cation intermixing as a major source of the interface 2DEG.

While the La in LAO exchanges predominantly with the Sr in STO, the Al exchanges differently with STO in the 3 u.c. and the 10 u.c. samples; Al predominantly replaces Ti through the Al ⇔ Ti site exchange in the 10 u.c. sample, but it can also replace Sr through the Al ⇔ Sr in the 3 u.c. sample (Figure S2). It is known that Al can in principle act as a donor or an acceptor in STO.[1] Calculation predicts that Al favorably replaces Sr in the insulating and/or oxygen deficient condition, but $\mathrm{Al}_{\mathrm{Ti}}'$ defects become increasingly favorable as the bulk condition becomes more *n*-type conductive and/or in the oxygen rich condition. Over a wide range of oxygen pressures Al will be amphoteric (forming both $\mathrm{Al}_{\mathrm{Sr}}^{\bullet}$ and $\mathrm{Al}_{\mathrm{Ti}}'$), resulting in Al self-compensation. The occurrence of Al ⇔ Sr site exchange in the 3 u.c. sample also supports the interface being insulating.

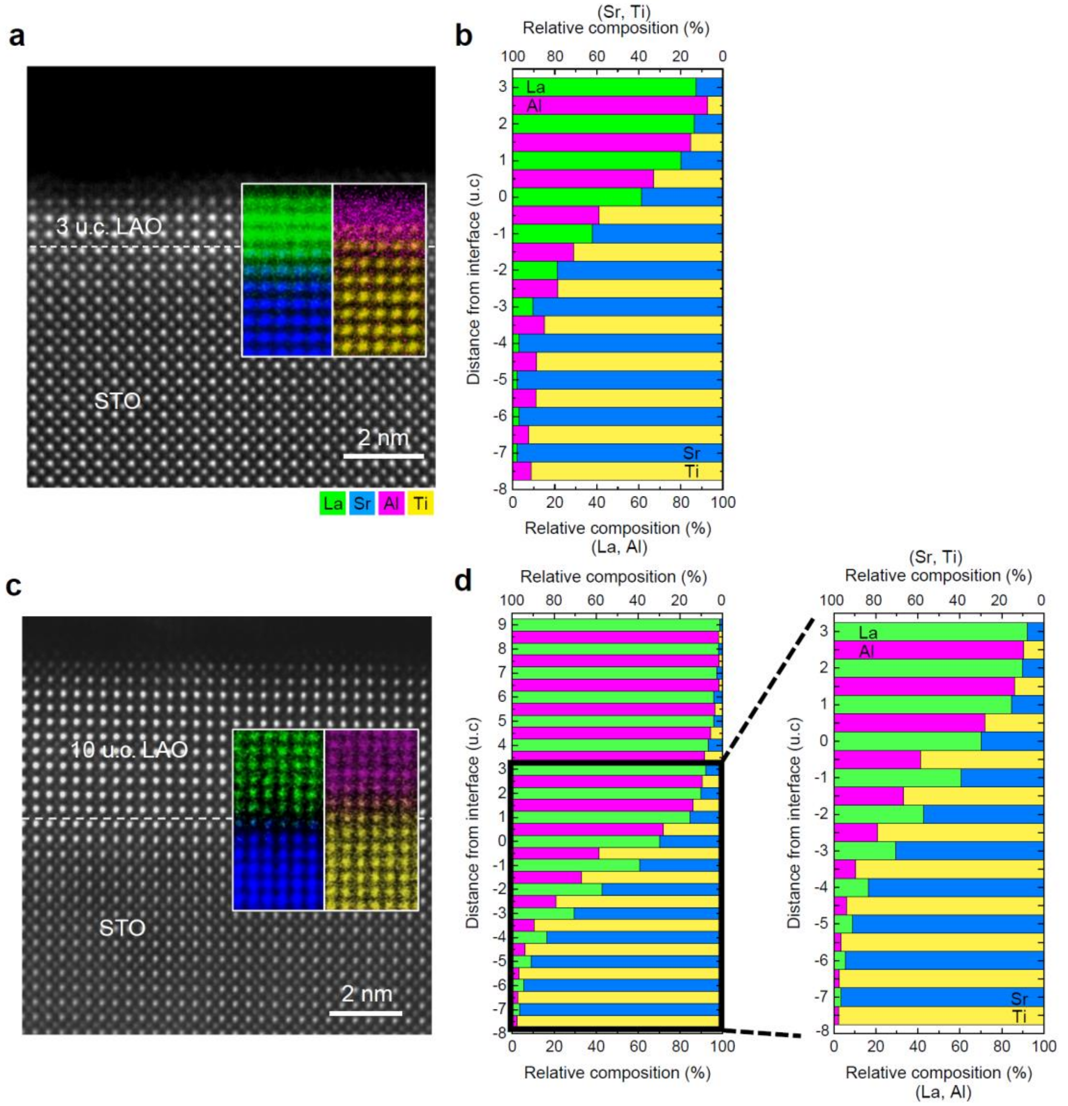

Figure S1. Atomic-column resolved STEM EDS elemental maps and profiles. a), c) EDS elemental maps constructed by selecting La-L_α and Sr-L_α for A-site cations and Al-K_α and Ti-K_α for B-site cations are overlaid on STEM HAADF image of the 3 u.c. and the 10 u.c. LAO/STO, respectively. The dashed line marks the nominal interface. b), d) Relative atomic ratio of cations in each layer of LAO/STO across the interaface determined by quantification of EDS map. The cation intermixing occurred in exchange between the cations at the same sites, i.e. A-site exchange (La ⇔ Sr) and B-site exchange (Al ⇔ Ti) across the interface. The extent of cation intermixing is similar in both 3 u.c. and 10 u.c. LAO/STO heterostructures. The maximum mixing appears in the first u.c. of each layer. After the first u.c. from the interface the intermixing drops rapidly below 20% and the composition of each material becomes stoichiometric in the subsequent layers. The Al ⇔ Sr site exchange was also noticed for some of the insulating 3 u.c. samples (Figure S2).

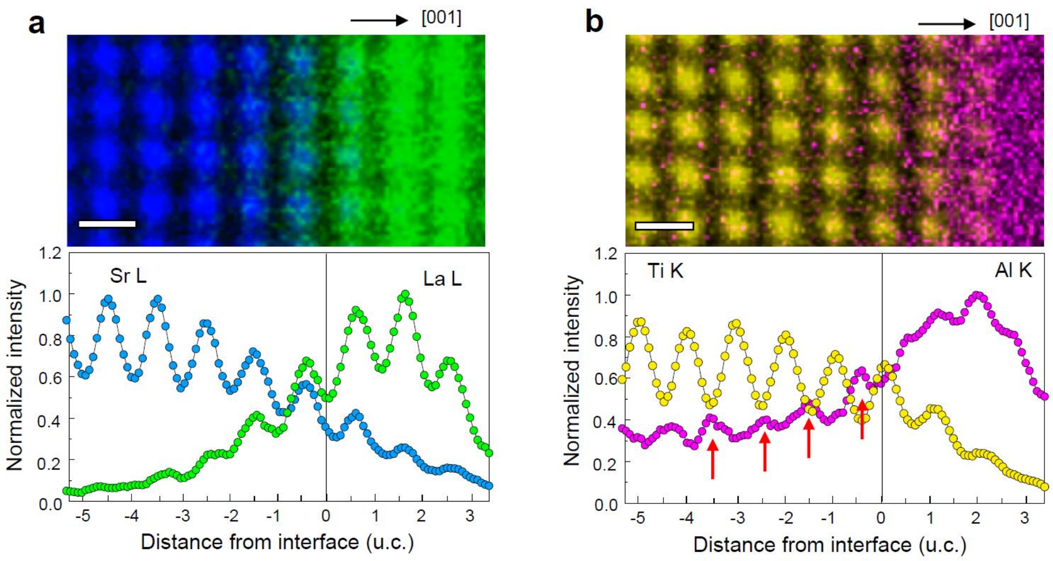

Figure S2. Atomic-column resolved STEM EDS elemental maps and composition profiles of 3 u.c. LAO/STO (001). a), b) EDS elemental map and corresponding normalized intensity profiles of A-site cations and B-site cations, respectively. While the La in LAO exchanges predominantly with the Sr in STO, the Al also replaces Sr through Al ⇔ Sr as indicated by red arrows in (b). The EDS elemental mapping was performed by the multiple frame summation up to less than 400 frames with the 256 × 256 pixels resolution and an acquisition time of 10 μs per pixel. Background noise floor in each map was removed by applying a weak Wiener filter. Scale bar is 5 Å.

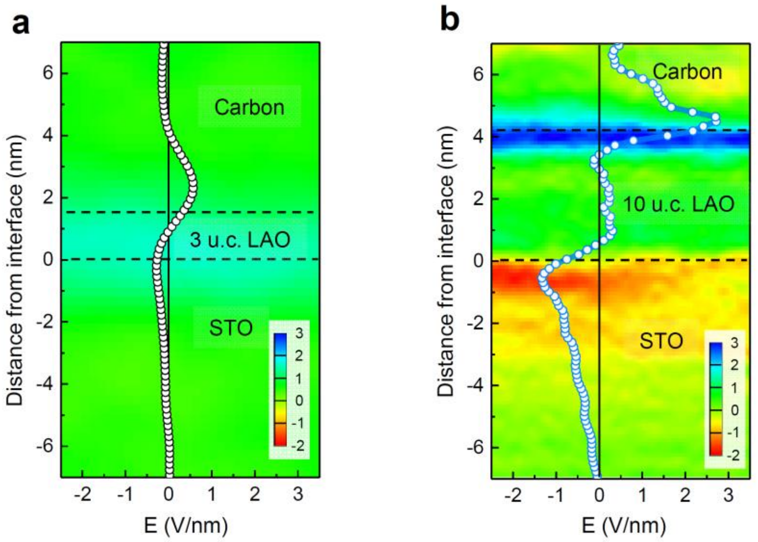

Figure S3. Electric field map obtained by inline electron holography. a), b) Electric field map measured by inline electron holography for 3 u.c. and 10 u.c. LAO/STO sample, respectively. Electric field map was obtained by taking negative derivative of a thickness-calibrated potential map. The averaged profile of the out-of-plane field component (E_y) of the electric field vector, $\mathbf{E} = (E_x, E_y)$, was overlaid on the electric field map. The electric field inside LAO film was negligibly small in both samples. For the 10 u.c. sample, large local fields with opposite signs were observed at the LAO surface and the LAO/STO interface.

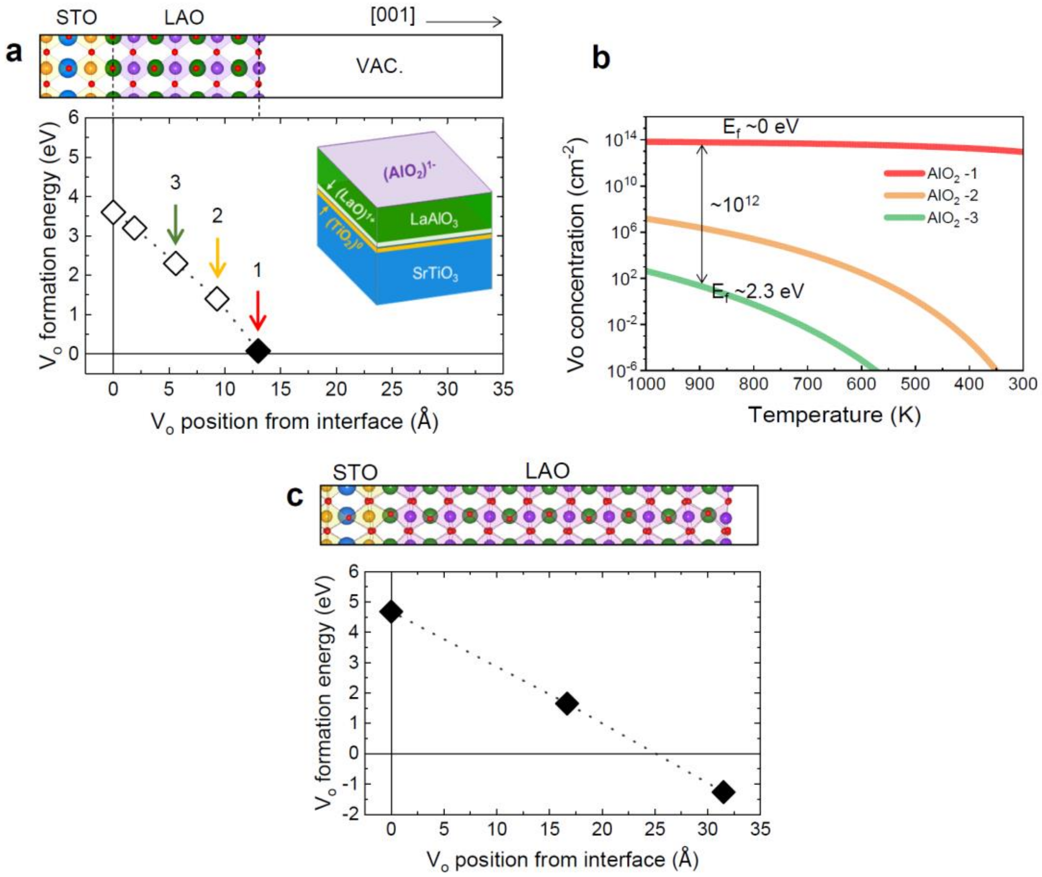

Figure S4. Formation energy of V_O in LAO/STO heterostructure. a) Formation energy of V_O at various locations in 4 u.c. LAO/STO heterostructure and b) the corresponding concentration of V_O as a function of temperature, respectively. The concentration of V_O was calculated following the Arrhenius type equation, such as $n_{VO} = N_{VO}\exp(-E_f / k_b T)$, where N_{VO} is the V_O concentrations required to neutralize the internal field in LAO, corresponding to $0.5/a^2$, E_f is the formation energy calculated by DFT, k_b is the Boltzmann constant, and T is the temperature. c) Formation energy of V_O at various locations in 9 u.c. LAO/STO heterostructure.

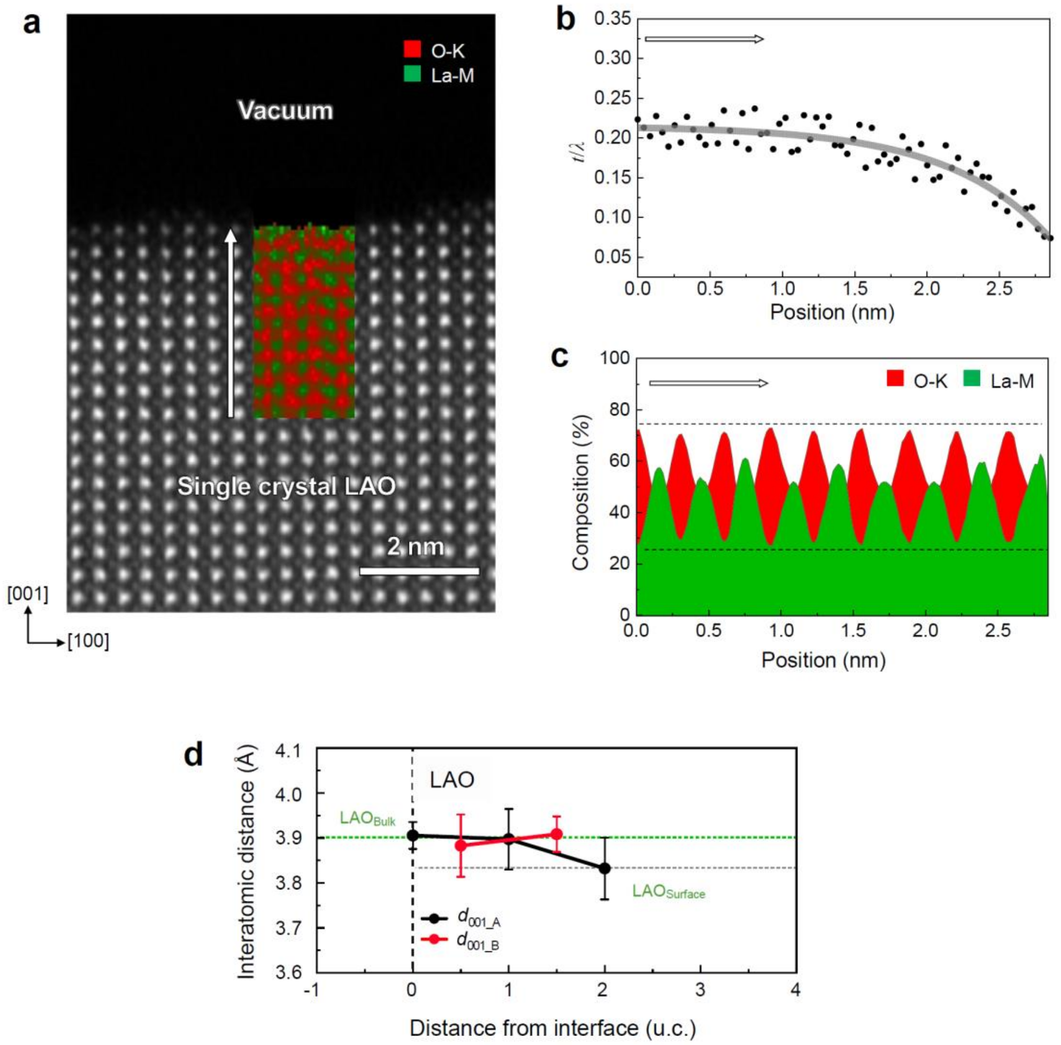

Figure S5. EELS results showing the stoichiometric O/La ratio preserved at LAO (001) single crystal surface without V_O. a) STEM HAADF image of atomically flat and clean LAO (001) surface. La (green) and O (red) elemental map obtained from EELS spectrum image is superimposed. The LAO (001) surface is terminated by LaO layer. b) Thickness profile in the t/λ scale extracted from low-loss EELS spectrum image, where t is the thickness and λ is the mean free path for inelastic scattering. The grey line is a parabolic fit to the t/λ profile. c) Atom-resolved composition profile obtained by EELS quantification using La-$M_{4,5}$ (green) and O-K (red) edges. The result shows that the O/La ratio remains stoichiometric at 3.0 without oxygen deficiency even though TEM sample thickness gradually decreases toward the surface. d) Plot of the A-site lattice constant (d_{001_A}) and B-site lattice constant (d_{001_B}) along the [001] out-of-plane direction. The surface relaxaiton behavior of single crystal LAO (001) surface, i.e., the contraction of the first surface unit cell, is consistent with the typical surface relaxation of LAO/STO (001) heterostructure in the absence of V_O except for the different surface termination, further supporting the absence of V_O at the LAO (001) surface.

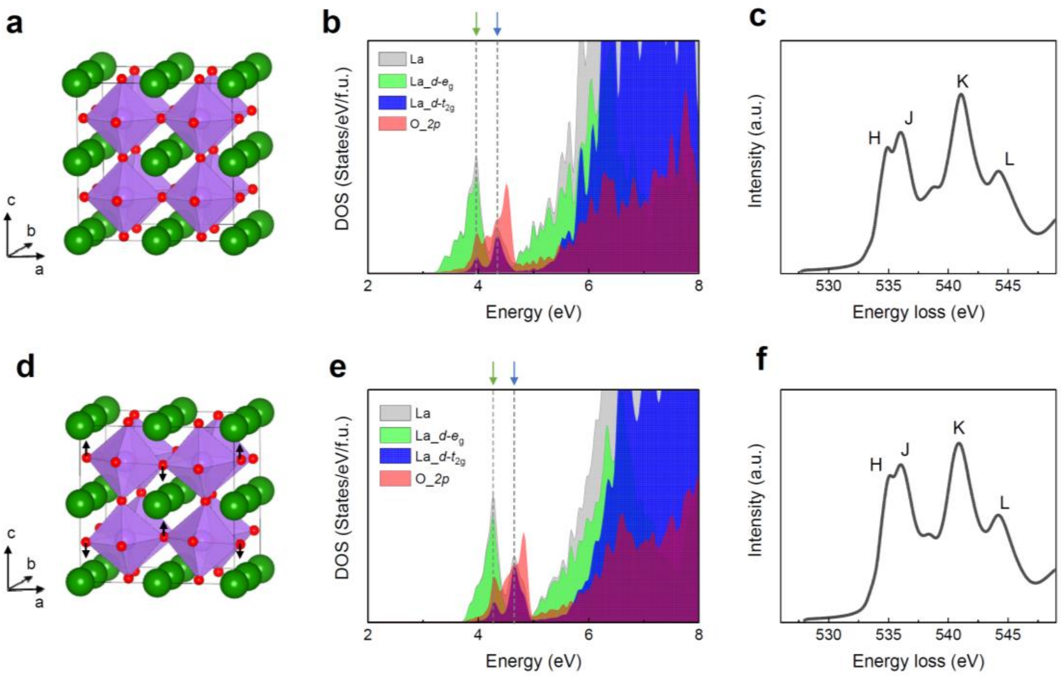

Figure S6. Effect of AFD rotation on projected density of states (PDOS) and EELS O-K edge. a) Simulation cell of LAO without AFD rotation. b), c) Calculated PDOS and EELS O-K edge by DFT. d) Simulation cell of LAO with AFD rotation ($a^-a^-a^-$), and the corresponding calculation result of e) PDOS and f) EELS O-K edge. The t_{2g}–e_g orbital splitting of La-*d* orbital was observed in PDOS, and peak splitting of O-2*p* orbital also appeared in the same energy level. In EELS O-K edge, the peaks H and J originate from the transition from O 1*s* to the hybridized O 2*p* and La 5*d* states, which are separated by ~1.7 eV due to the these t_{2g}–e_g crystal-field splitting. These calculations show that the presence of octahedral rotation does not affect the PDOS and EELS significantly.

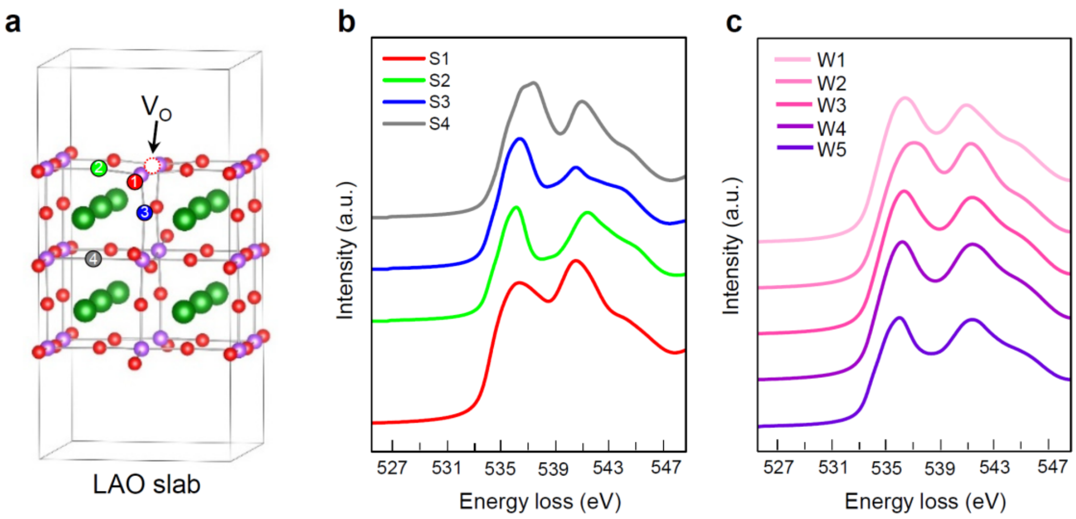

d

Weighted O-K edge	O-K edge of four oxygen atoms around the V_O			
	S1	S2	S3	S4
W1	50%		50%	
W2	50%			50%
W3	30%	30%	25%	15%
W4	40%	40%	20%	
W5	60%	30%	10%	

Figure S7. Calculation of EELS O-K edge by DFT calculation. a) Simulation cell of LAO for the calculation of EELS O-K edge by DFT. One V_O was introduced to the surface AlO_2 layer of a 2×2×2 supercell. b) Calculated EEL spectra of O-K edge for the oxygen at four different sites (indicated as 1, 2, 3 and 4) with respect to the vacancy position. c) Weighted average of four EEL spectra (S1, S2, S3 and S4 in (b)) with different weighting rates. The five different EEL spectra (W1-W5) were considered with various weighting rates to determine the best fit to the experimental STEM EELS data. Unequal weighting was given to each EEL spectrum depending on the distance from the V_O. The balanced weighting that takes account of the distance from the V_O (W5), e.g. 0.6, 0.3, 0.1 and 0 for the oxygen atom at 1, 2, 3 and 4, respectively, yielded the best fit. d) Weighting rates assigned to the four EEL spectra (S1, S2, S3, and S4 in (b)) to prepare the EEL spectra W1-W5 shown in (c).

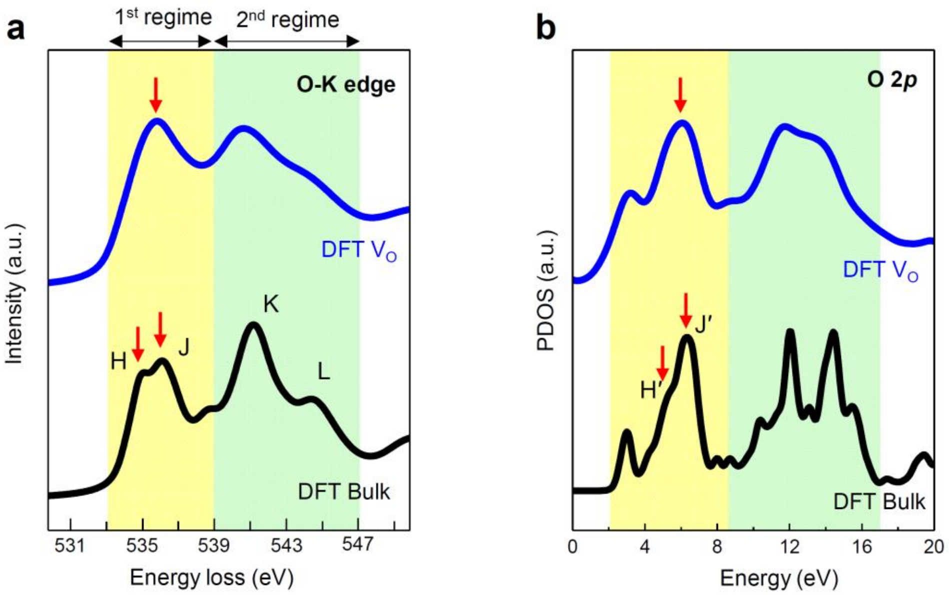

Figure S8. Calculated EELS O-K edges and projected density of states (PDOS) of O 2*p* by DFT. a) EELS O-K edge calculated using DFT (CASTEP code) for bulk LAO (black line) and LAO slab with V_O (blue line). The blue line is the best fit obtained by weighted average of the EELS O-K edges calculated for the four different oxygen sites near the surface V_O (W5 in Figure S7c). b) Calculated oxygen density of states (O 2*p* PDOS) for bulk LAO (black line) and LAO slab with V_O (blue line). For bulk LAO, two peaks (H, J and H', J' for O-K edge and O 2*p* PDOS, respectively) are separated by the t_{2g}-e_g crystal-field splitting in the octahedral coordination as indicated by red arrows. In the presence of V_O, however, the two peaks merged into a single peak, which indicates that the V_O broke the octahedral symmetry by changing the relative distance between La and O atoms, and then modified the hybridization between La and O 2*p* states.

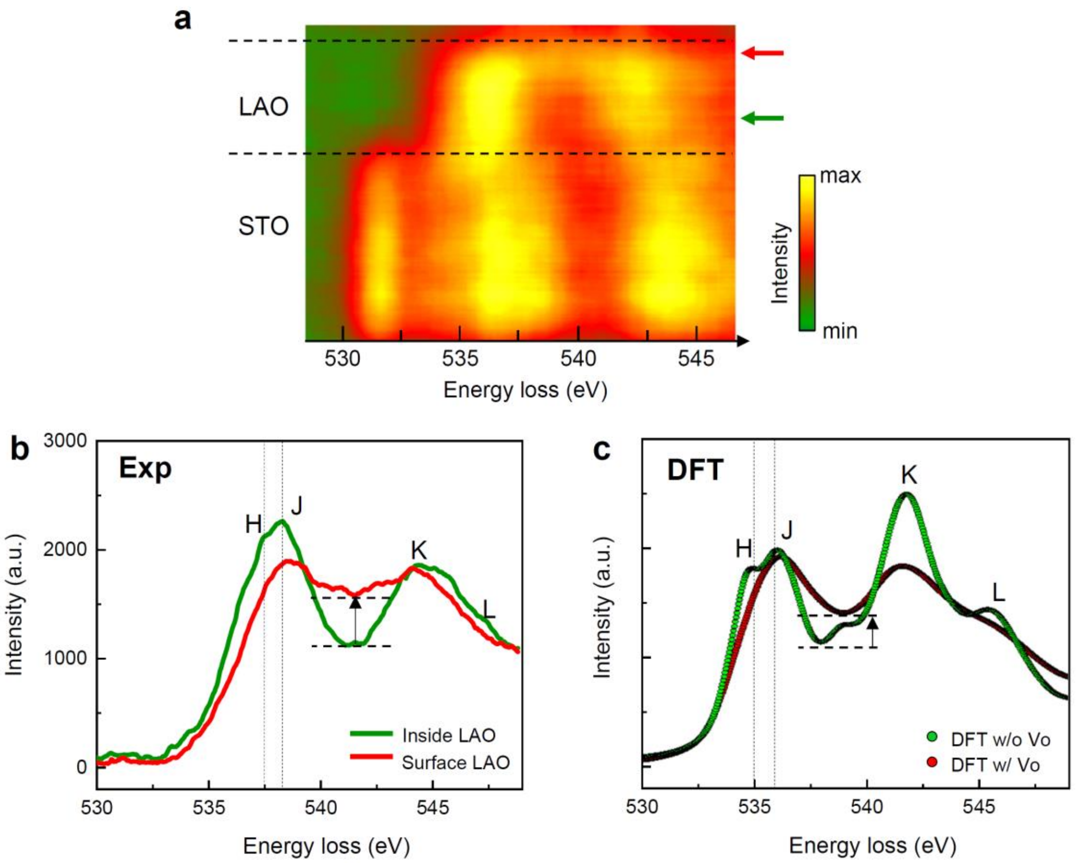

Figure S9. EELS O-K edges measured by EELS line scan. a) 2D visualization of EELS line scan. b) STEM EELS O-K edge from the 10 u.c. LAO/STO (001) heterostructure. The averaged O-K edges from the middle of LAO (green) and from the LAO surface (red) are compared. c) Simulated O-K edges of LAO slab without (green) or with (red) V_O.

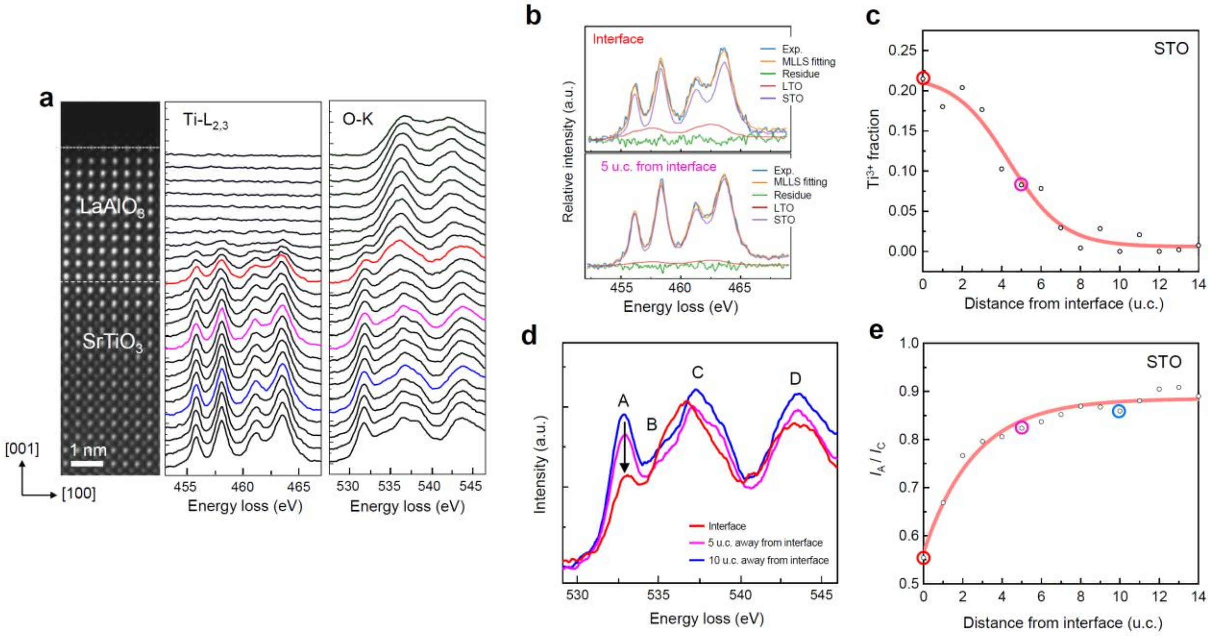

Figure S10. STEM EELS Ti-$L_{2,3}$ and O-K edge profile of 10 u.c. LAO/STO, revealing the confinement of 2DEG at Ti-3*d* orbitals in the absence of V_O at the STO side of interface. a) STEM HAADF image and EELS line profiles of Ti-$L_{2,3}$ and O-K edges from STO substrate to LAO surface. b) Multiple linear least square (MLLS) fitting of Ti-$L_{2,3}$ edge to determine the relative fraction of Ti^{3+} from Ti^{4+}. With an assumption that the Ti-$L_{2,3}$ edge is composed of a linear combination of Ti^{4+} and Ti^{3+}, their relative weight fraction was determined by fitting the experimental Ti-$L_{2,3}$ edge using the two reference spectra, a purple one for Ti^{4+} ($SrTiO_3$, STO) and a red one for Ti^{3+} ($LaTiO_3$, LTO).[3-5] c) Ti^{3+} fraction measured by MLLS plotted as a function of distance from interface. The orange line represents the best fit to the experimental spectrum. d) Comparison of EELS O-K edges extracted from different locations in the STO i.e., the interfacial TiO_2 plane (red line), 5 u.c. (magenta line) and 10 u.c. (blue line) away from the interface. Suppression of peak A at the interfacial TiO_2 plane is indicated by black arrow. e) Plot of the intensity ratio of peak A and C (I_A/I_C) as a function of distance from the interface. A decreasing I_A/I_C near the interface is due to the existence of Ti^{3+} and the lack of V_O.[6] A high fraction of Ti^{3+} and lower I_A/I_C value at the STO side of the interface demonstrate the presence of 2DEG but the absence of V_O.

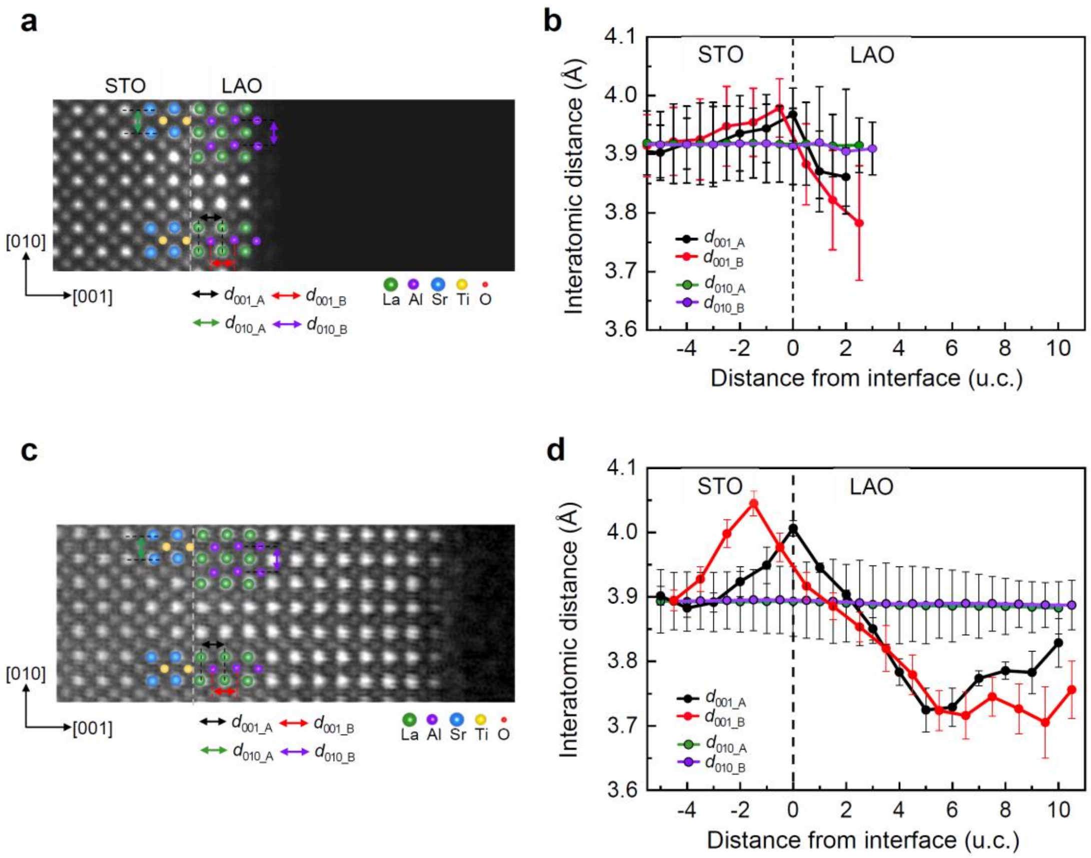

Figure S11. Lattice constants of LAO/STO heterostructures measured by STEM HAADF. a), b) STEM HAADF images taken at the [100] zone axis and plot of the A-site lattice constant and B-site lattice constant along the [010] in-plane direction and [001] out-of-plane direction for 3 u.c. sample. c), d) STEM HAADF images taken at the [100] zone axis and plot of the A-site lattice constant and B-site lattice constant along the [010] in-plane direction and [001] out-of-plane direction for 10 u.c. sample. Each data point is an average over 30 u.c. along the [010] in-plane direction. The error bars represent the standard deviation.

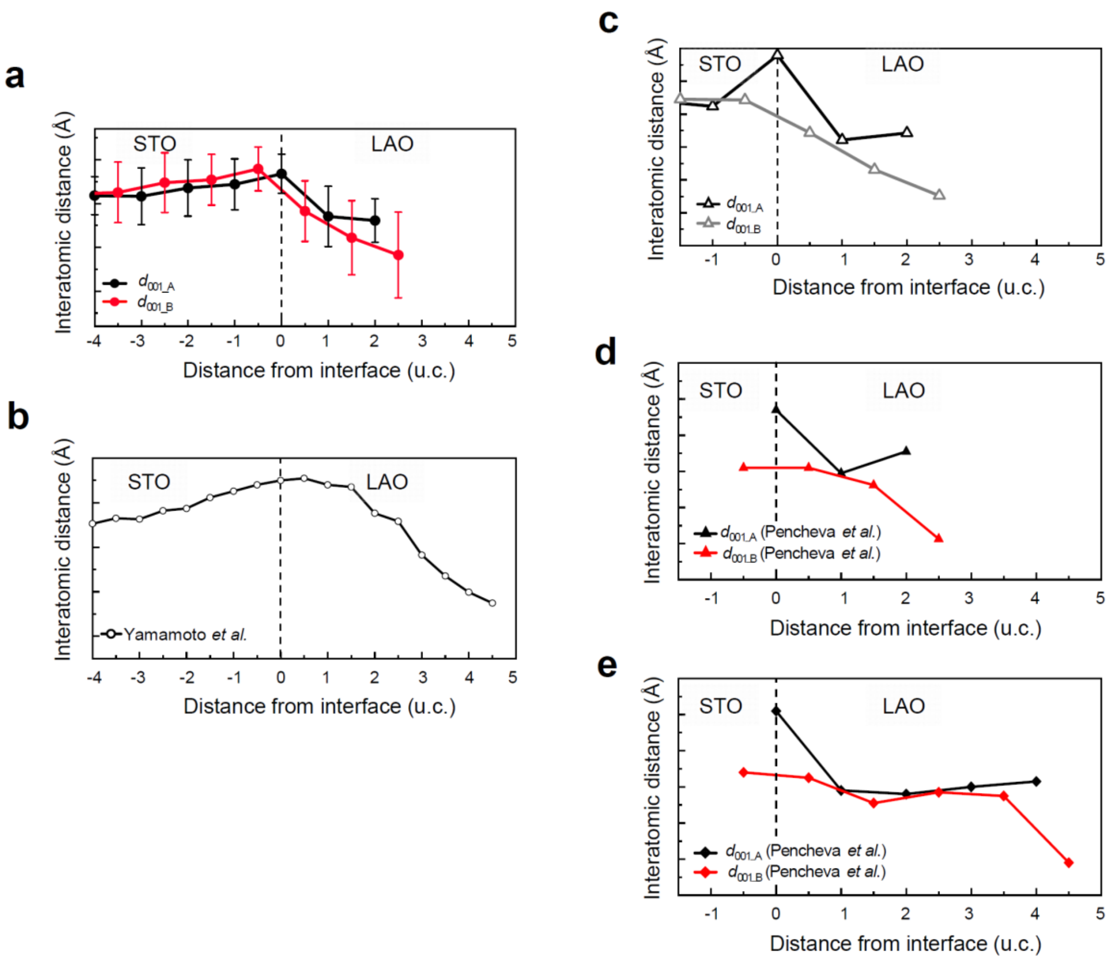

Figure S12. Unit cell-resolved lattice constants of LAO/STO heterostructure – Comparison of experimental measurements and DFT calculations a) Experimental measurement of 3 u.c. LAO/STO heterostructure based on STEM images in the present study. b) Experimental measurement of 5 u.c. LAO/STO heterostructure based on X-ray diffraction by Yamamoto et al.[7] Both a) and b) consistently show the characteristic lattice constant variation across the LAO/STO interface. c) DFT calculation of 3 u.c. LAO/STO heterostructure in the present study. DFT calculation of d) 3 u.c. and e) 5 u.c. LAO/STO heterostructure by Pentcheva and Pickett.[8] All DFT calculations consistently show the characteristic lattice constants at the LAO surface, where the La atoms in the sub-surface LaO layer are displaced toward the surface, leading to the expansion of the A-site cation lattice constant, but the B-cation lattice constant is contracted due to the movement of Al atom in the surface AlO_2 layer toward the interface.

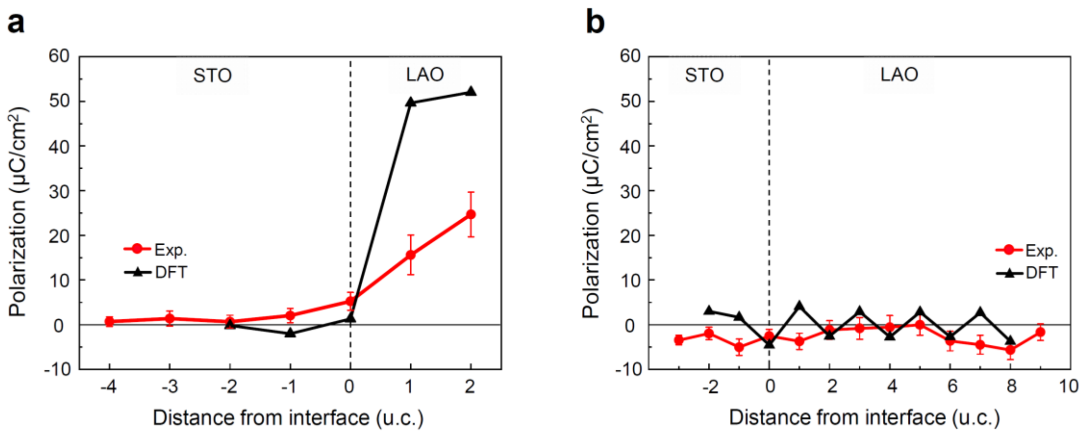

Figure S13. Net dipole moment per unit cell (polarization) meaured from STEM ABF images. a), b) Polarization measured from the atomic displacements shown in Figure 4d and Figure 5d for the 3 u.c. and the 10 u.c. LAO/STO sample, respectively. For the 3 u.c. sample, the positive polarization is induced by FE polar displacements, which predominantly compensates the built-in polar field. On the contrary, for the 10 u.c. sample the polarization induced by atomic displacement is almost zero, in consistent with the compensation of the polar field predominantly by 2DEG formation. Each polarization value was obtained by averaging over 15 u.c. along the [110] in-plane direction. The error bars represent the standard deviation. The dash line marks the nominal interface.

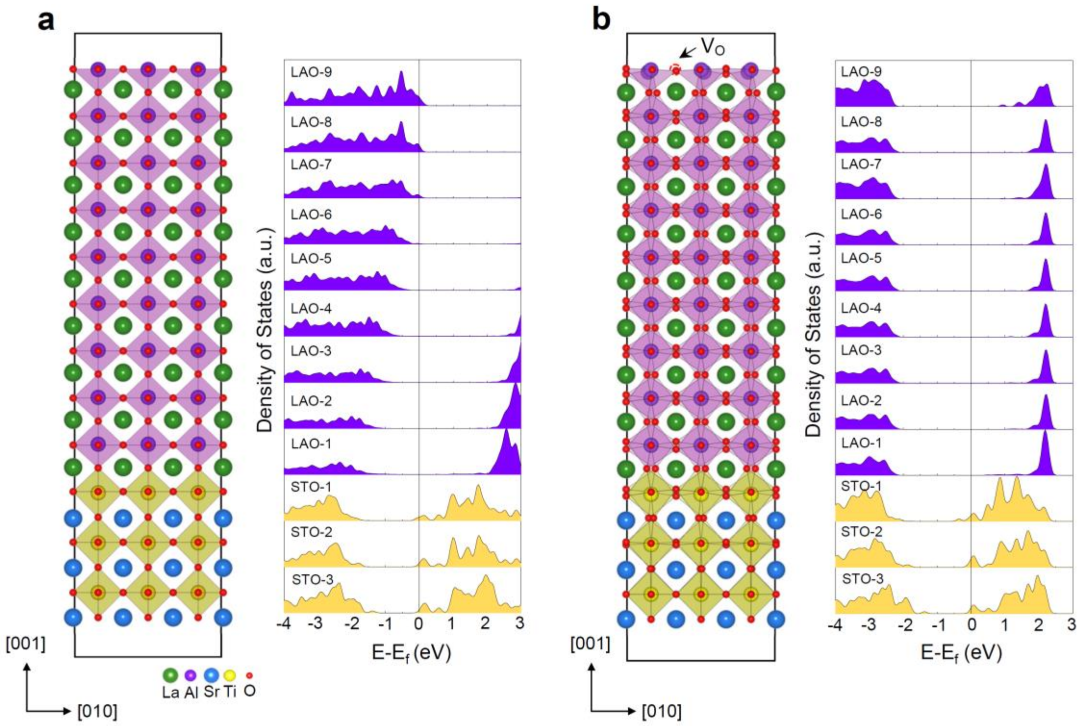

Figure S14. Layer-projected DOS in LAO/STO supercell calculated by DFT. Structural model of LAO/STO and layer-projected DOS for 2×2 $(LAO)_9/(STO)_4$ (*n*-type interface) slabs a) without V_O and b) with V_O on the LAO surface, respectively. For LAO/STO slab without V_O on the LAO surface, as the density of the transferred electron is less than 0.5 *e*/Å, internal polar field inside LAO layer is partially compensated, resulting in upward band-shift from the interface to the LAO surface. On the contrary, for LAO/STO slab with V_O on the LAO surface internal polar field inside LAO layer is completely compensated, resulting in a straight alignment of the valence band maximum of LAO layers.

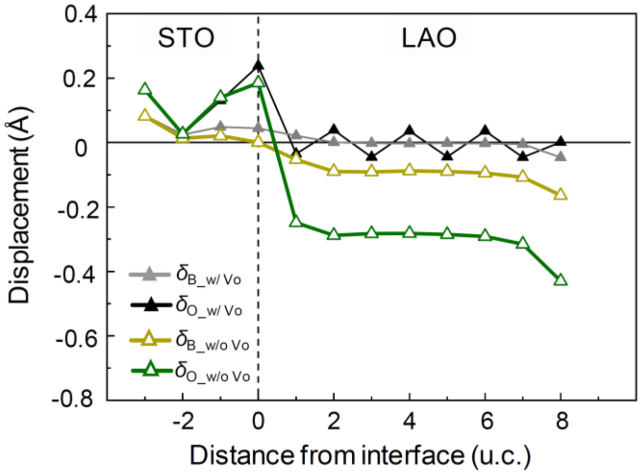

Figure S15. Atomic displacements of LAO/STO calculated by DFT. Plot of the atomic displacements of B-site cations (δ_B) and O atoms (δ_O) measured by DFT results without V_O (hollow triangle) and with V_O on the LAO surface (filled triangle), respectively. The atomic displacements of B-site cations were determined by measuring the distance δ_B from the center-of-mass (COM) position of the A-site cations. The displacements of O atoms (δ_O) were determined by measuring the shift of averaged O atom position belonging to the BO_2 plane from the COM of A-site cations. The large displacement of O atom measured at the STO side of interface generates the negative buckling,[9] which arises mainly from the electrostrictive effects due to the local fields associated with confined 2DEG and Ti^{3+} valence state.

Reference

[1] L. Yu, A. Zunger, *Nat. Commun*. **2014**, *5*, 5118.

[2] S. A. Chambers, *Sur. Sci.* **2011**, *605*, 1133.

[3] A. Ohtomo, D. A. Muller, J. L. Grazul, H. Y. Hwang, *Appl. Phys. Lett.* **2002**, *80*, 3922.

[4] D. A. Muller, N. Nakagawa, A. Ohtomo, J. L. Grazul, H. Y. Hwang, *Nature* **2004**, *430*, 657.

[5] N. Nakagawa, H. Y. Hwang, D. A. Muller, *Nat. Mater*. **2006**, *5*, 204.

[6] C. Cantoni, J. Gazquez, F. Miletto Granozio, M. P. Oxley, M. Varela, A. R. Lupini, S. J. Pennycook, C. Aruta, U. S. di Uccio, P. Perna, D. Maccariello, *Adv. Mater*. **2012**, *24*, 3952.

[7] R. Yamamoto, C. Bell, Y. Hikita, H. Y. Hwang, H. Nakamura, T. Kimura, Y. Wakabayashi, *Phys. Rev. Lett.* **2011**, *107*, 036104.

[8] R. Pentcheva, W. E. Pickett, *Phys. Rev. Lett.* **2009**, *102*, 107602.

[9] S. A. Pauli, S. J. Leake, B. Delley, M. Bjorck, C. W. Schneider, C. M. Schleputz, D. Martoccia, S. Paetel, J. Mannhart, P. R. Willmott, *Phys. Rev. Lett.* **2011**, *106*, 036101.
